# Macrophages, but not neutrophils, are critical for proliferation of *Burkholderia cenocepacia* and ensuing host-damaging inflammation

**DOI:** 10.1371/journal.ppat.1006437

**Published:** 2017-06-26

**Authors:** Jennifer Mesureur, Joana R. Feliciano, Nelly Wagner, Margarida C. Gomes, Lili Zhang, Monica Blanco-Gonzalez, Michiel van der Vaart, David O’Callaghan, Annemarie H. Meijer, Annette C. Vergunst

**Affiliations:** 1 VBMI, INSERM, Univ. Montpellier, Nîmes, France; 2 Institute of Biology Leiden, Leiden University, Leiden, The Netherlands; Duke University, UNITED STATES

## Abstract

Bacteria of the *Burkholderia cepacia* complex (Bcc) can cause devastating pulmonary infections in cystic fibrosis (CF) patients, yet the precise mechanisms underlying inflammation, recurrent exacerbations and transition from chronic stages to acute infection and septicemia are not known. Bcc bacteria are generally believed to have a predominant extracellular biofilm life style in infected CF lungs, similar to *Pseudomonas aeruginosa*, but this has been challenged by clinical observations which show Bcc bacteria predominantly in macrophages. More recently, Bcc bacteria have emerged in nosocomial infections of patients hospitalized for reasons unrelated to CF. Research has abundantly shown that Bcc bacteria can survive and replicate in mammalian cells *in vitro*, yet the importance of an intracellular life style during infection in humans is unknown. Here we studied the contribution of innate immune cell types to fatal pro-inflammatory infection caused by *B*. *cenocepacia* using zebrafish larvae. In strong contrast to the usual protective role for macrophages against microbes, our results show that these phagocytes significantly worsen disease outcome. We provide new insight that macrophages are critical for multiplication of *B*. *cenocepacia* in the host and for development of a fatal, pro-inflammatory response that partially depends on Il1-signalling. In contrast, neutrophils did not significantly contribute to disease outcome. In subcutaneous infections that are dominated by neutrophil-driven phagocytosis, the absence of a functional NADPH oxidase complex resulted in a small but measurably higher increase in bacterial growth suggesting the oxidative burst helps limit bacterial multiplication; however, neutrophils were unable to clear the bacteria. We suggest that paradigm-changing approaches are needed for development of novel antimicrobials to efficiently disarm intracellular bacteria of this group of highly persistent, opportunistic pathogens.

## Author summary

*Burkholderia cenocepacia* is a bacterial pathogen that increases morbidity and mortality in cystic fibrosis patients, but can also cause serious hospital-acquired infections. In this work we demonstrate that macrophages, but not neutrophils, critically contribute to the development of a rapidly fatal inflammatory infection caused by *B*. *cenocepacia*. In contrast to findings with other important human pathogens, including *Mycobacterium* and *Salmonella*, Bcc bacteria cannot replicate in zebrafish embryos depleted of macrophages, identifying macrophages as a critical site for bacterial replication *in vivo*. Our study sheds light on the contribution of infected macrophages to infection outcome in an animal model, and contributes a paradigm changing concept of the infection mechanism of this class of bacterial pathogens.

## Introduction

Bacteria belonging to the *Burkholderia cepacia* complex (Bcc) are ubiquitously found in the natural environment, specifically the rhizosphere of plants, and were first identified as the cause of onion rot; however, they are important opportunistic pathogens of individuals with cystic fibrosis (CF) and chronic granulomatous disease (CGD) [1]. More recently, they have emerged in nosocomial infections of both immunocompromised and immunocompetent patients hospitalized for reasons unrelated to CF or CGD; these infections have been correlated with contaminated surfaces and central venous access [2–4], but not much is known about reasons for the pathological effects. Multiple resistance to most clinically used antibiotics complicates treatment strategies [1,2].

In CF patients, pulmonary disease is the most important cause of morbidity and mortality. Vicious cycles of pulmonary obstruction, chronic microbial infections, and ineffective airway inflammation result in progressive deterioration of lung function [5]. The disease is characterized by massive neutrophil infiltration and production of pro-inflammatory cytokines, including IL-1β, TNFα and the neutrophil chemo attractant CXCL8 (IL8). Infections with Bcc bacteria aggravate clinical outcome with recurrent exacerbations and an increased pro-inflammatory state, and often result in fatal necrotizing pneumonia and septicemia known as Cepacia Syndrome. Neutrophils are fundamental in fighting infections, however, dysfunction of the cystic fibrosis transmembrane conductance regulator (CFTR) has been shown to result in an impairment in the ability of neutrophils to kill microbes and ineffective resolution of inflammation, which are considered as major causes of many of the pathological consequences seen in CF [6]. In *B*. *cenocepacia*-colonized CF patients neutrophil activation has been suggested to play an important role in the observed clinical deterioration [7]. In addition, human neutrophils have been shown to be able to reduce *B*. *cenocepacia* numbers in a reactive oxygen species (ROS)-dependent manner *in vitro* [8], contributing the increased sensitivity of CGD patients towards Bcc bacteria to a dysfunctional NADPH oxidase and the inability to produce an oxidative burst. Currently, no information is available about a role for neutrophils during nosocomial non-CF infections.

It has been demonstrated extensively using mammalian cell culture models that *B*. *cenocepacia* can build an intracellular niche and evade host immune killing [reviewed in 9–11]. In macrophages, *B*. *cenocepacia* has been shown to interfere with the phagolysosomal maturation process, membrane trafficking and the autophagy machinery [11–13]. Macrophages from individuals with CF or CGD have been shown to exhibit enhanced susceptibility towards *B*. *cenocepacia* infection, which has been attributed to reduced acidification and phagolysosomal fusion, defective autophagy and lower autophagic flux, respectively [12–14]. *B*. *cenocepacia* has also been detected in macrophages of experimentally infected rodents and zebrafish larvae [15–17]. While *in vitro*, macrophages stimulated with LPS or infected with *B*. *cenocepacia* display a significantly increased cytokine production [18–20], the direct contribution of Bcc-infected macrophages to pro-inflammatory state and disease outcome *in vivo* is not known.

Here we use a zebrafish (*Danio rerio*) infection model, taking advantage of the high similarity of its immune system to that of humans [21], and the excellent possibilities of intravital imaging of transparent zebrafish embryos that have contributed to better understanding microbe/host phagocyte interactions [22,23]. We demonstrate that macrophages are critical for proliferation of *B*. *cenocepacia* in zebrafish embryos. Moreover, we show that infection with *B*. *cenocepacia* leads to fatal inflammation requiring Il1-dependent signalling, and that Bcc bacteria can escape neutrophil defences. We discuss the implications of our findings in the context of development of therapeutic strategies to control Bcc infections.

## Results

### Proliferation of *B*. *cenocepacia* in the host and ensuing fatal infection critically depend on macrophages

Upon intravenous (iv) injection in zebrafish embryos, bacteria belonging to the Bcc are phagocytosed mainly by macrophages, in which they can replicate [17]. To better understand the explicit role of macrophages during infection with Bcc bacteria we used two strategies to deplete macrophages from zebrafish embryos; knockdown of pu.1 [24], a transcription factor involved in early myeloid progenitor formation, and chemical ablation.

**Fig 1:**
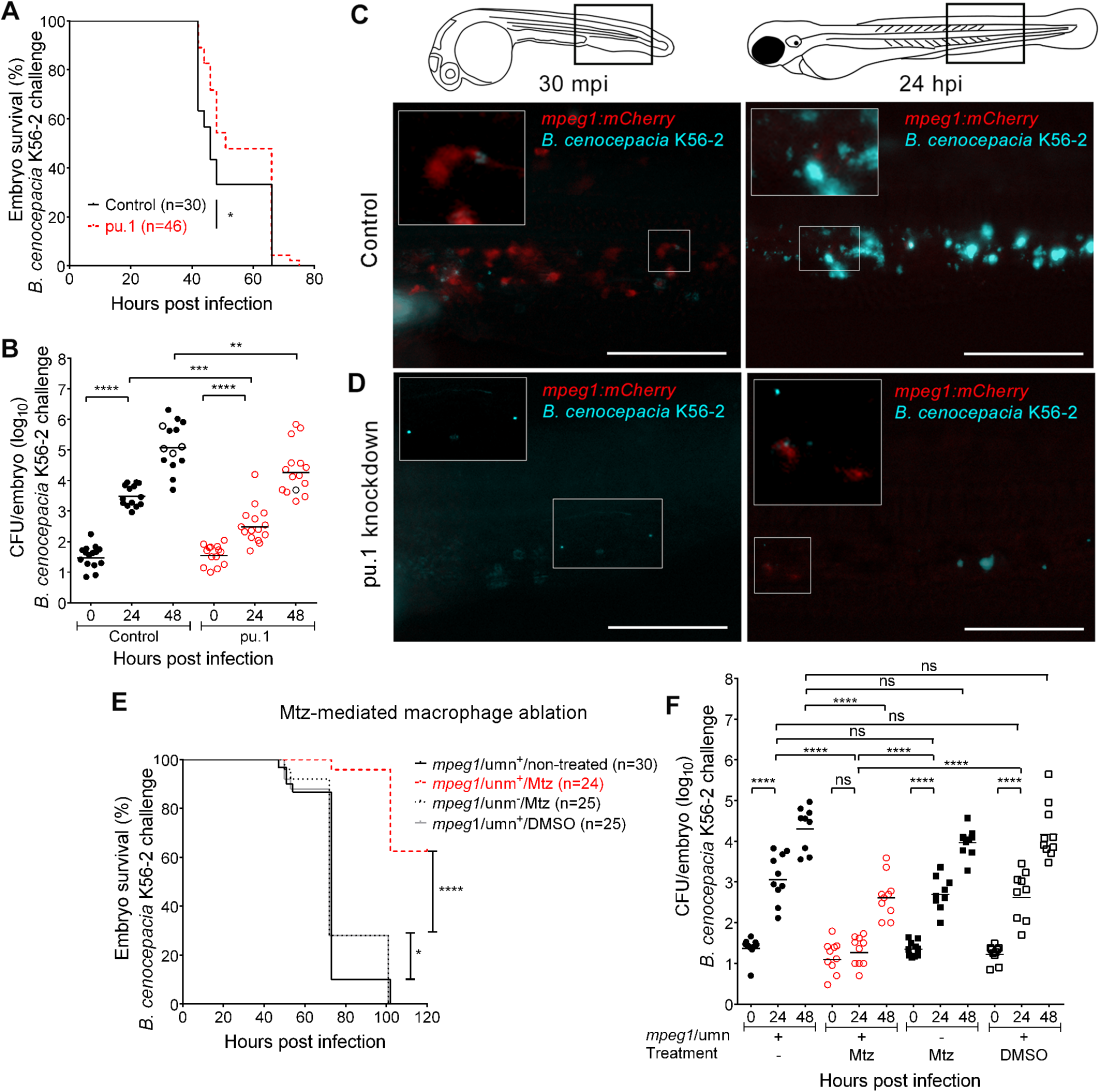
Macrophages are critical for virulence of *B*. *cenocepacia*. **(A,B)** Embryo survival (average inoculum 17 CFU, representative experiment) **(A)** and bacterial burden (total of 3 experiments) over time **(B)** of control (black) and pu.1 knockdown embryos (red) injected iv with *B*. *cenocepacia* K56-2. **(C,D)** Representative fluorescence overlay images of an *mpeg1*:*mCherry* control and *mpeg1*:*mCherry* pu.1 knockdown embryo at 30 min and 24 h after injection with ~40 CFU *B*. *cenocepacia* K56-2 (blue). See also S2 Fig. **(C)** mCherry-positive macrophages (red) colocalise with K56-2 at 30 mpi, and are no longer detected at 24 hpi (insets show magnification). **(D)** mCherry-positive macrophages are absent in knockdown embryos at 30 mpi and start to re-appear at 24 hpi (insets show magnification). Scale bars, 100 μm. **(E,F)** Embryo survival (average inoculum 28 CFU, representative experiment) **(E)** and corresponding bacterial burden (n = 10 per group per time point). **(F)** of *mpeg1*/umn^+^ embryos, untreated or treated with 5mM Mtz or 0.2% DMSO, and *mpeg1*/umn^−^embryos treated with 5mM Mtz iv injected with *B*. *cenocepacia* K56-2. **(B,F)** Geometric means with each data point representing an individual embryo. Dead embryos marked as black open circles (not recorded for 5 embryos in **(B)**, and in **(F**)). **(A, B, E, F)** * p ≤ 0.05, ** p ≤ 0.01, *** p ≤ 0.001; **** p ≤ 0.0001. ns: not significant. See materials and methods for statistical tests. See also S1 Fig and S3 Fig.

*B*. *cenocepacia* K56-2, J2315 or *B*. *cepacia* CEP509, epidemic strains that we have previously shown to cause rapidly fatal systemic infection in zebrafish embryos [17], surprisingly showed a significant reduction in host mortality after depleting embryos of macrophages by pu.1 knockdown (Fig 1A, S1A Fig, S1C Fig and S1E Fig). Reduced mortality correlated with reduced bacterial burden in pu.1 knockdown compared to control embryos (Fig 1B, S1B Fig and S1D Fig). Intravital imaging of iv injected embryos, which express mCherry in macrophages (*Tg(mpeg1*:*mCherry-F)*, referred to as *mpeg1*:*mCherry*, or *Tg(mpeg*:*Gal4/uas-mCherry-NTR)*, referred to as *mpeg1*/umn) also showed that pu.1 knockdown embryos contained fewer bacteria at 24 hours post infection (hpi) than control embryos (Fig 1C and 1D and S2 Fig), demonstrating that macrophages are critical for bacterial replication. The applied concentration of pu.1 morpholino resulted in specific depletion of macrophages (Fig 1D compared to Fig 1C, 30 mpi), but not neutrophils (S1F Fig), although the effect was temporary as newly formed mCherry-positive macrophages appeared during infection (Fig 1D, 24 hpi and S2 Fig). In control embryos, the bacteria were rapidly seen associated with macrophages as shown previously [17], and the mCherry-positive signal was found to colocalise with bacteria up to 16 hpi; however, the mCherry positive signal had disappeared completely in the heavily infected control embryos, suggesting macrophages disappeared during the later stages of acute infection (Fig 1C and S2 Fig, and see below). The data suggest that an intramacrophage replication stage is critical for an increase in bacterial burden and for progression to rapidly fatal infection.

Next, we adopted a chemically-inducible targeted cell ablation strategy based on the metronidazole/nitroreductase (Mtz/NTR) system [25]. Treatment of *mpeg1/*umn^+^ zebrafish embryos, which express the *mCherry-NTR* fusion protein in macrophages (S3A Fig), with the prodrug Mtz resulted in efficient specific ablation of fluorescent macrophages (S3B Fig and S3C Fig). Although DMSO- and Mtz-treated non-fluorescent *mpeg1*/umn^-^ embryos (see materials and methods) showed slightly reduced bacterial load and mortality compared to non-treated infected control embryos (Fig 1E and 1F), Mtz-treated *mpeg1*/umn^+^ embryos lived significantly longer after iv injection of *B*. *cenocepacia* K56-2, with up to 60% of the embryos still alive at 5 days post infection (Fig 1E). Strikingly, no significant multiplication of *B*. *cenocepacia* was seen in macrophage-depleted embryos up to 24 hpi, in contrast to the infected control embryos (Fig 1F). The dependence on macrophages for replication was confirmed by real time imaging of infected DMSO- and Mtz-treated *mpeg1*/umn^+^ larvae (S4 Fig). Together, these data strengthen the pu.1 knockdown results, and demonstrate that macrophages are critical for multiplication of iv injected *B*. *cenocepacia* K56-2, J2315 and *B*. *cepacia* CEP509 and for the development of fatal infection in zebrafish embryos.

### Macrophages clear less virulent Bcc strains inefficiently

**Fig 2:**
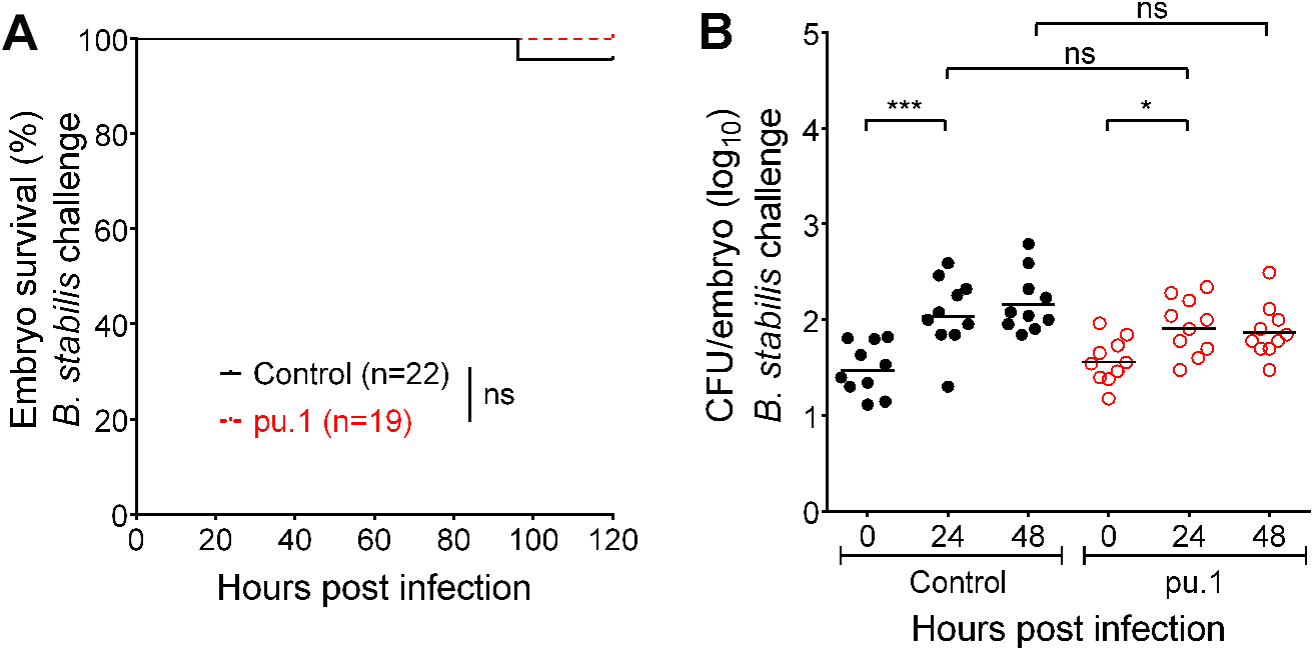
Macrophages clear less virulent Bcc strains inefficiently. **(A,B)** Embryo survival (average inoculum 23 CFU, representative experiment) **(A)** and bacterial burden (total of 2 experiments) over time **(B)** of control (black) and pu.1 knockdown embryos (red) iv injected with *B*. *stabilis* LMG14294. Geometric means with each data point representing an individual embryo. * p ≤ 0.05, *** p ≤ 0.001; ns: not significant. See materials and methods for statistical tests. See also S1 Fig.

Next, we determined the effect of macrophage depletion on the fate of three Bcc isolates that we have shown earlier to cause persistent infection in zebrafish embryos; iv-injected bacteria of these strains are able to survive and replicate in macrophages, yet they are unable to induce a pro-inflammatory response and disseminate from infected cells [17]. Pu.1 knockdown had no significant effect on host survival after infection with these strains (Fig 2A, S1G Fig and S1I Fig). For *B*. *stabilis* LMG14294 the absence of macrophages resulted in slightly but not significantly lower bacterial numbers at 48 hpi (Fig 2B), showing that, in agreement with the results described above for *B*. *cenocepacia*, these bacteria do not replicate efficiently in the absence of macrophages. *B*. *cenocepacia* J415 was cleared in 50% of the control, but not in pu.1 knockdown embryos at 48 hpi (S1H Fig), showing that macrophages have some protective role for the host against this strain. CFU counts were also higher at 48 hpi for *B*. *vietnamiensis* after pu.1 knockdown compared to control embryos (S1J Fig). Thus, although macrophages can reduce bacterial burden, they are unable to completely clear these less virulent Bcc strains, emphasizing the persistent character of this group of bacteria [17].

### Acute infection correlates with systemic phagocyte death

**Fig 3:**
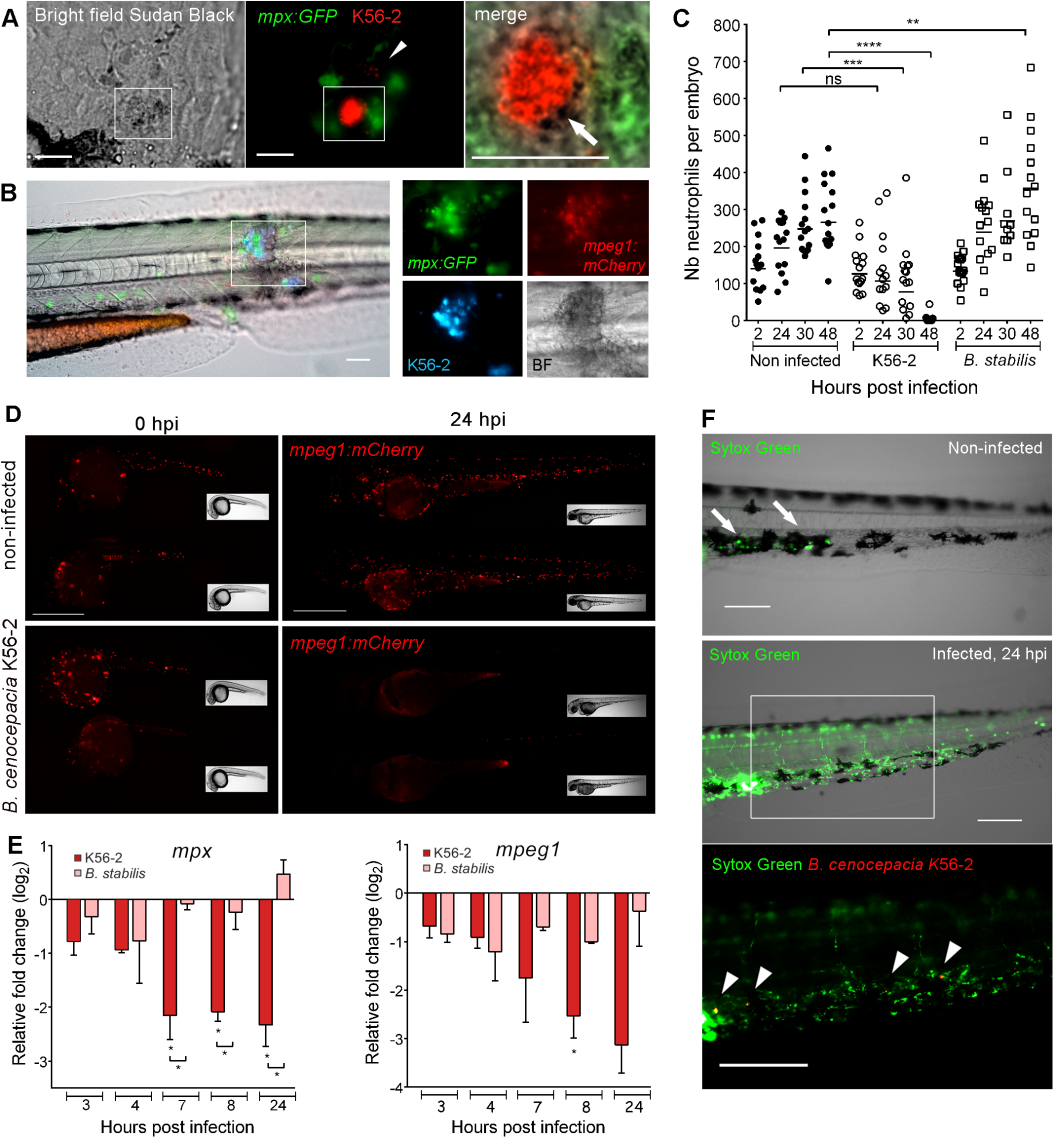
Acute, but not persistent infection results in systemic phagocyte death. **(A)** Sudan black staining of an *mpx*:*GFP* embryo (24 hpi), injected with ~45 CFU *B*. *cenocepacia* K56-2. Bright field, fluorescence and merged images showing recruited neutrophils (green) that release granules (stained by Sudan black as black deposit, white arrow) close to an infected cell containing red fluorescent bacteria. Arrow head, individual bacteria. Scale bars, 50 μm. **(B)** Image of the trunk region of an *mpx*:*GFP*; *mpeg1*:*mCherry* embryo 24 h post iv injection in the blood island with *B*. *cenocepacia* K56-2 (Turquoise), showing neutrophils (green) and macrophages (red) infiltrated in an infection site with multiple infected cells. BF, Bright field image, showing tissue damage. Scale bar, 50 μm. **(C)** Mean neutrophil numbers in non-infected control and *mpx*:*GFP* embryos injected at 50 hpf with *B*. *cenocepacia* K56-2 or *B*. *stabilis* LMG14294. See also S5B Fig and S5C Fig. **(D)** *Mpeg1*:*mCherry* embryos showing reduced macrophage numbers (red) at 24 hpi in *B*. *cenocepacia* K56-2-infected (~45 CFU) compared to non-infected control embryos. Scale bars, 0.5 mm. See S5D Fig for quantification. **(E)** Mean relative *mpx* and *mpeg1* gene expression level (qRT-PCR) in embryos injected with on average 234 CFU of *B*. *cenocepacia* K56-2 (red bars) or 123 CFU of *B*. *stabilis* LMG14294 (pink bars) each normalised to a PBS-injected control group at each time point and analysed using Anova (error bars, SEM). Two independent experiments. Asterisks below each bar indicate significance compared to the PBS control at each time point, significance between groups per time point is indicated with a horizontal line. **(F)** Non-infected and *B*. *cenocepacia* K56-2 (~50 CFU, Turquoise indicated in red for better visualization) infected embryos at 24hpi with the cell-impermeable dye Sytox Green. Arrows, dead cells due to DMSO injection. Arrow heads, bacterial clusters. Scale bars, 100 μm. See also S5F Fig. **(C).** Each data point represents an individual embryo. **(C,E)** * p ≤ 0.05; ** p ≤ 0.01; *** p ≤ 0.001; **** p ≤ 0.0001; ns: non-significant. See materials and methods for statistical tests. See also S5 Fig.

In contrast to macrophages, neutrophils do not efficiently phagocytose Bcc bacteria injected in the circulation [17]. Here, we analysed their behaviour during later stages after iv injection in *mpx*:*GFP* embryos. After uptake and replication of *B*. *cenocepacia* in macrophages, patrolling neutrophils often completely stretched around these infected cells without apparent success in eliminating them (S5A Fig). From this time point, recruited neutrophils were seen to degranulate close to infected cells which might contribute to the release of intracellular bacteria, although the mechanism for bacterial release or exit from infected macrophages is not clear (Fig 3A). The infection progressed, as described earlier [17] with the formation of infection sites throughout the body, defined as areas of local bacterial dissemination, repeated cycles of intracellular replication, and tissue damage. Here, we show that these infection sites are characterized by massive infiltration of both macrophages and neutrophils (Fig 3B). During acute infection caused by *B*. *cenocepacia* K56-2, GFP-positive neutrophils (Fig 3C) and mCherry-positive macrophages (Figs 1C and 3D and S5D Fig) rapidly disappeared from the whole embryo body, including the infection sites. In contrast, an increase in neutrophil numbers was observed in a percentage of the embryos during persistent infection with *B*. *stabilis*, and recruited neutrophils were only occasionally seen to accumulate near infected macrophages (Fig 3C, S5B Fig and S5C Fig).

Global expression analysis showed that the neutrophil-specific *mpx* and macrophage-specific *mpeg1* genes were rapidly downregulated in embryos injected with K56-2, but not with *B*. *stabilis* (Fig 3E). The observed disappearance of fluorescent phagocytes during infection with *B*. *cenocepacia* K56-2, however, was not simply due to reduced expression levels of the *mpx*:*GFP* and *mpeg1*:*mCherry* transgenes; labelling of the leukocyte-specific protein L-plastin or with the macrophage-specific dye Neutral Red [26] also revealed drastically reduced phagocyte numbers in infected embryos, although infected macrophages still stained positive for L-plastin (S5E Fig). Importantly, staining with the cell impermeable dye SYTOX Green showed that K56-2-infected embryos were loaded with extracellular DNA, in contrast to few SYTOX-positive cells at the site of injection in non-infected embryos (Fig 3F). Systemic lysis of macrophages was confirmed in infected *mpeg1/*umn^+^ embryos, which show strong expression of mCherry in macrophages allowing visualization of their destruction as mCherry-positive debris (S4 Fig (24–48 hpi; DMSO-treated) and S5F Fig). Thus, excessive inflammation correlates with systemic neutrophil and macrophage death although infected macrophages stay intact.

**Fig 4:**
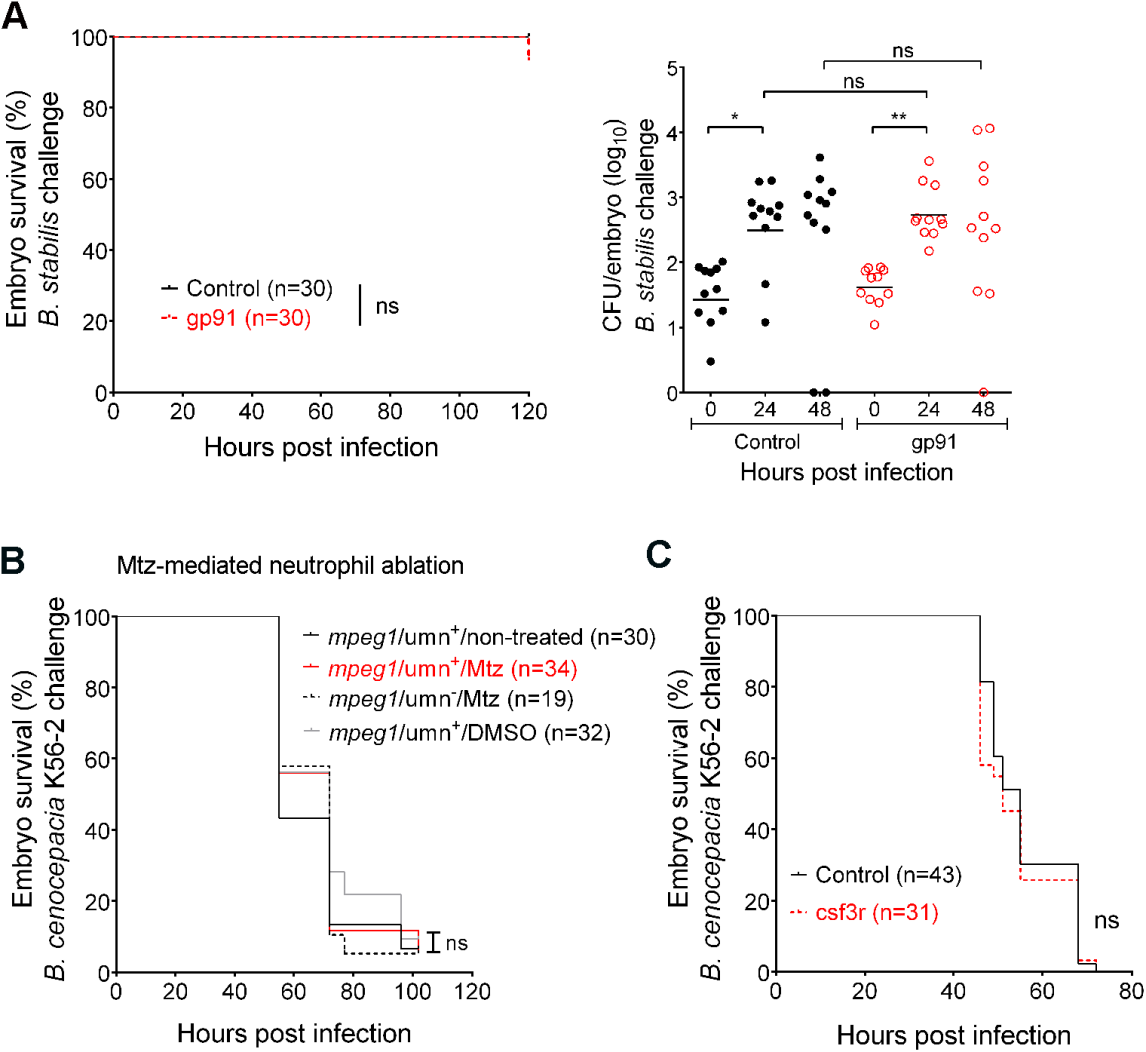
Neutrophils do not contribute significantly to infection. **(A)** Embryo survival (left; representative experiment) and bacterial burden (total of 2 experiments) over time (right panel, geometric mean) of control (black) and gp91 knockdown embryos (red) iv injected with *B*. *stabilis*. Each data point represents an individual embryo. **(B)** Embryo survival of *mpx*/umn^+^ embryos, untreated or treated with 10 mM Mtz or 0.2% DMSO, and *mpx*/umn^−^embryos treated with 10 mM Mtz iv injected with ~ 50 CFU *B*. *cenocepacia* K56-2. **(C)** Embryo survival (average inoculum 50 CFU, representative experiment) of control (black) and csf3R knockdown embryos (red) injected iv with *B*. *cenocepacia* K56-2. * p ≤ 0.05; ** p ≤ 0.01; ns: non-significant. See materials and methods for statistical tests.

Neutrophils constitute a major source of ROS during inflammation. To analyse whether the production of ROS contributes to disease outcome, we performed knockdown experiments of the *gp91*^*phox*^ (or *cybb*) subunit of NADPH oxidase [27]. Knockdown of gp91^phox^ did not significantly affect embryo survival and bacterial burden in embryos iv injected with *B*. *cenocepacia* K56-2, J2315 or *B*. *stabilis* LMG14294 (Fig 4A), suggesting ROS-mediated responses do not effectively contribute to host defence, or host-damaging inflammation.

To study whether neutrophils themselves contributed to the observed tissue damage and host mortality we depleted embryos from neutrophils in analogy to the chemical ablation of macrophages described above. We obtained fish lines expressing the *mCherry-NTR* fusion protein in neutrophils and pre-incubated *mpx*/umn^+^ larvae with Mtz or DMSO. No difference was observed after iv injection with *B*. *cenocepacia* K56-2 in non-treated, DMSO-treated, or Mtz-treated *mpx*/umn^-^ larvae compared to Mtz-treated *mpx*/umn^+^ larvae (Fig 4B), showing that the absence of neutrophils does not significantly change disease outcome. In agreement with these results, knockdown of csf3R, which results in specific neutrophil ablation [28], did not affect embryo survival rates (Fig 4C). The finding that depletion of neutrophils did not have a net effect on host survival emphasizes the host-destructive role of macrophages during acute infection caused by *B*. *cenocepacia*.

### Neutrophils rapidly phagocytose, but are unable to eradicate, subcutaneously introduced Bcc

**Fig 5:**
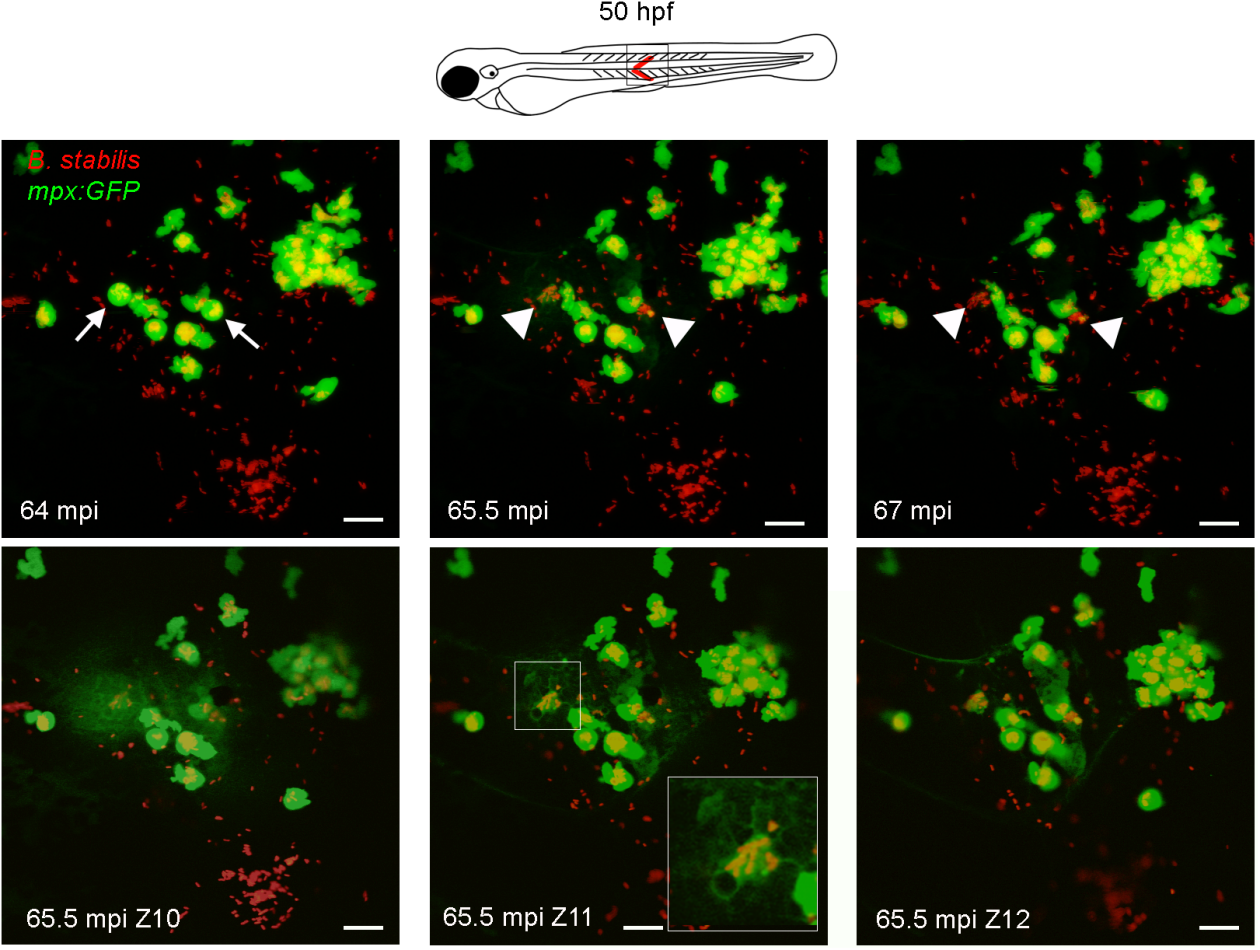
Neutrophils efficiently phagocytose surface-associated Bcc bacteria. Confocal stacks after subcutaneous infection with *B*. *stabilis* in *mpx*:*GFP* embryos (STD intensity projection, 2 μm x 19 steps; T = 35–37 in S2 Movie). Arrows, rounded neutrophils (green) with vacuoles full of bacteria (red) at 64 minutes post injection (mpi) eject their cell contents in the surroundings (arrow heads 65.5 mpi), leaving bacterial clusters and cell debris (arrow heads, 66 min). Lower panels, MAX intensity projection of three consecutive slices (2 μm) at 65.5 mpi showing ejected cellular contents (diffuse GFP signal). Scale bars, 50 μm.

Injection of Bcc in the circulation of embryos is marked by macrophage-dominated phagocytosis [17] and our data now show that neutrophils do not significantly contribute to the fatal outcome of these infections. To better understand the role of neutrophils upon phagocytosis of bacteria we performed experiments based on the elegant work by Colucci-Guyon and colleagues who showed that neutrophils efficiently sweep up surface-associated but not fluid-borne bacteria [29]. Intravital imaging of *mpx*:*GFP* embryos subcutaneously injected with *B*. *cenocepacia* K56-2 or *B*. *stabilis* LMG14294 showed that neutrophils were rapidly attracted to live and heat-killed bacteria, but not to PBS, and infiltrated the infected area (S1 Movie and S2 Movie). Surprisingly, after about 1 h we observed that both *B*. *cenocepacia* K56-2 and *B*. *stabilis*-containing GFP-positive neutrophils rounded up and spontaneously ejected their cell contents into the extracellular space covering a large area (Fig 5, S2 Movie), although this was not evident with lower infection doses (S1 Movie). The released bacteria remained clustered and other neutrophils in the area showed reduced mobility directly after the event (S2 Movie).

**Fig 6:**
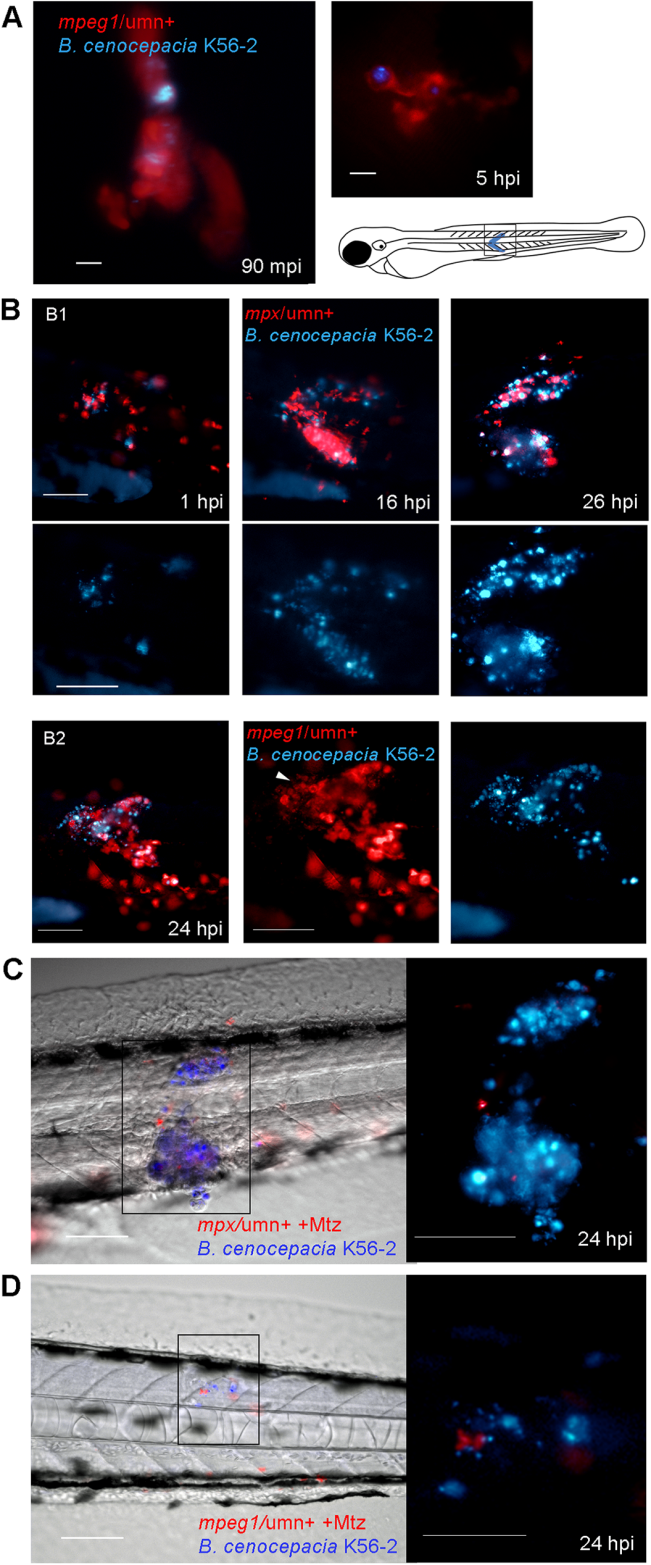
Macrophages, but not neutrophils, contribute to increased bacterial burden and pro-inflammatory responses towards subcutaneously introduced *B*. *cenocepacia*. **(A)** *Mpeg1/*umn^+^ embryos were subcutaneously injected with *B*. *cenocepacia* K56-2 expressing Turquoise. Fluorescent overlay images were taken at 90 min and 5 h post infection, showing infected macrophages (red). Scale bars, 10 μm. **(B)** Images (red and blue overlay and below slightly enlarged individual fluorescence images with blue filter) of the indicated area (see drawing) of an *mpx/*umn^+^ **(B1)** and an *mpeg1/*umn^+^ **(B2)** embryo subcutaneously injected with *B*. *cenocepacia* K56-2 expressing Turquoise. B1 shows an embryo followed in time displaying neutrophil infiltration (red) and increase in bacterial burden (blue, see Fig 7A for quantification). B2 shows the image of an embryo with macrophage infiltration (red) and high bacterial burden at 24 hpi. Arrow head points at mCherry positive debris. See non-infected *mpx/*umn^+^ and *mpeg1/*umn^+^ control embryos in S6 Fig for comparison. Scale bars, 100 μm. **(C)** Image of the infected area of a representative *mpx/*umn^+^ embryo depleted of neutrophils with Mtz, and subcutaneously injected with *B*. *cenocepacia* K56-2 (Turquoise) at 24 hpi. Scale bar 100 μm, and 50 μm for inset. See also S6D Fig. **(D)** Image of a representative *mpeg1/*umn^+^ embryo depleted of macrophages with Mtz and subcutaneously injected with *B*. *cenocepacia* K56-2 (Turquoise) at 24 hpi. Scale bar 100 μm, and 50 μm for inset.

**Fig 7:**
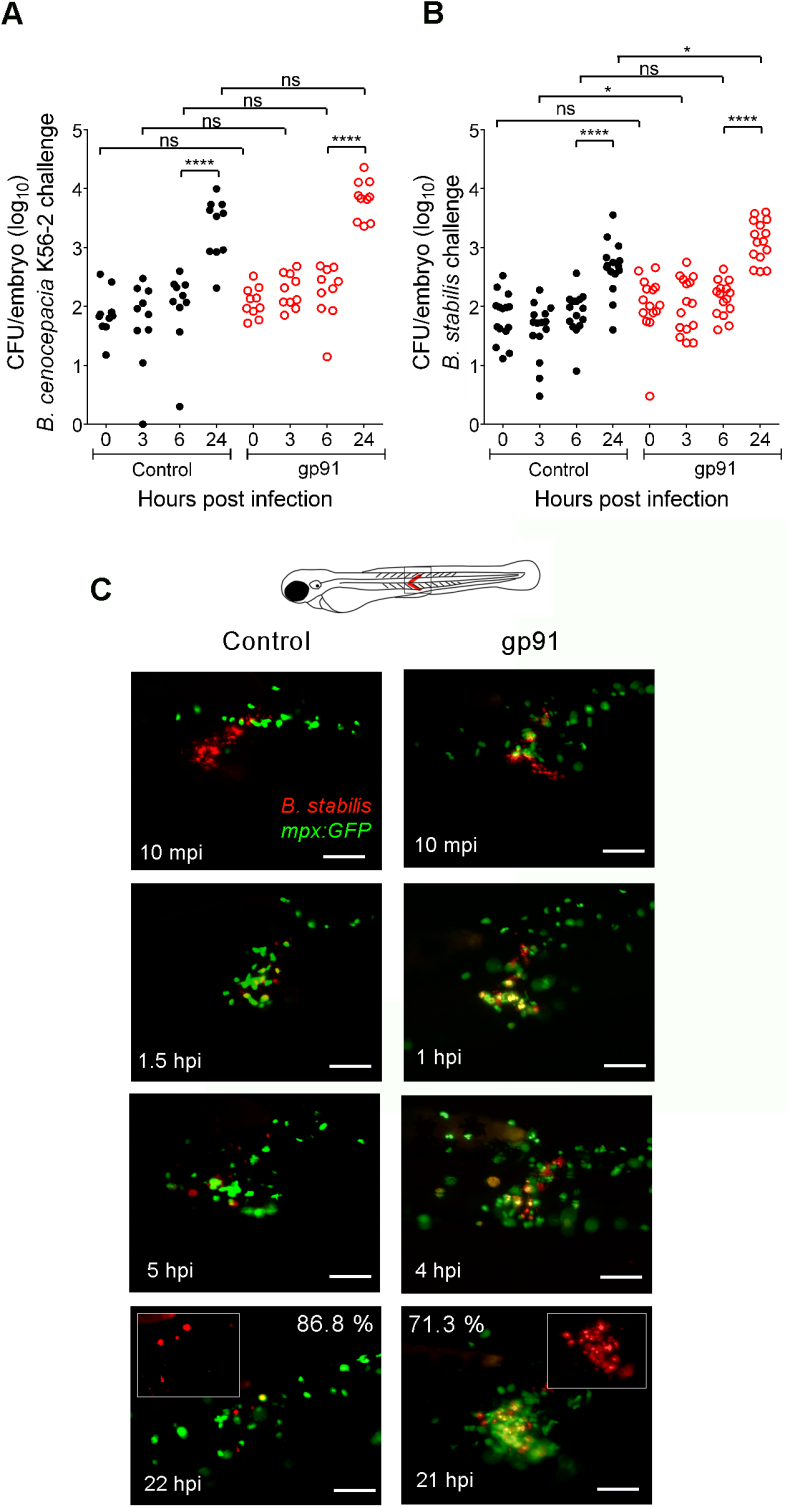
The absence of Gp91 results in increased bacterial burden and neutrophil persistence during subcutaneous infections. **(A)** Bacterial burden over time after subcutaneous injection of *B*. *cenocepacia* K56-2 in control (black circles) and gp91 knockdown embryos (open red circles). Average of two independent experiments. **(B)** Bacterial burden over time after subcutaneous injection of *B*. *stabilis* LMG14294 in control (black circles) and gp91 knockdown embryos (open red circles). Average of three independent experiments. **(C)** Fluorescent overlay images of the injected area of a representative *mpx*:*GFP* control MO and gp91 knockdown embryo (neutrophils in green) in time after subcutaneous injection with *B*. *stabilis* (red). Inset shows bacterial load at ~20 hpi. % at 22 and 21 hpi indicates percentage of control and gp91 knockdown embryos that show reduced neutrophil numbers (86.8%), and persistent neutrophil infiltration (71.3%), respectively, at the infection site. Scale bar, 100 μm. **(A,B)** * p ≤ 0.05; **** p ≤ 0.0001; ns: non-significant. See materials and methods for statistical tests.

Although the majority of subcutaneous bacteria were phagocytosed by recruited neutrophils (S1 Movie and S2 Movie), macrophages also contributed to bacterial uptake at the infection site, and bacterial clusters localised inside macrophages at later time points (Fig 6A and S6A Fig). Subcutaneous injection of *B*. *cenocepacia* K56-2 resulted in massive infiltration of neutrophils and macrophages, and was followed by visible tissue damage and an increase in bacterial burden with neutrophils persisting at the infection site in 100% of the infected embryos (Figs 6B and 7A, S6B Fig and S6C Fig). Subcutaneous injection of *B*. *stabilis* also resulted in an increase in bacterial burden, although reduced compared to *B*. *cenocepacia* (Fig 7B). In contrast to *B*. *cenocepacia* infection, only 13.2% (±6.85% SEM) of the embryos (3 independent experiments, n = 8, 12, and 26, respectively) showed persistent neutrophil infiltration at the infection site at 22 hpi, while neutrophils had moved away in the majority of embryos, suggesting that the host was able to resolve the inflammation resulting from infection with *B*. *stabilis* (Fig 7C, left panel).

To determine the individual contribution of neutrophils and macrophages during subcutaneous infections, *mpx*/umn^+^ and *mpeg1*/umn^+^ embryos depleted of neutrophils or macrophages, prior to infection using Mtz-mediated cell ablation, respectively, were subcutaneously injected with *B*. *cenocepacia* K56-2. The absence of neutrophils did not change bacterial multiplication (Fig 6C compared to Fig 6B). In subcutaneously injected Mtz-treated *mpeg1*/umn^+^ embryos, however, the absence of macrophages prevented bacterial multiplication (Fig 6D and S6D Fig). These data demonstrate that neutrophils are not able to control infection caused by subcutaneously injected *B*. *cenocepacia* K56-2, and that even upon neutrophil-dominated phagocytosis *B*. *cenocepacia* K56-2 is highly virulent through its interaction with macrophages.

Neutrophils of CGD patients are unable to mount an oxidative burst, and are less able to reduce Bcc numbers compared to those of healthy individuals [8,30]. Therefore, we analysed the effect of gp91 knockdown on subcutaneously injected *B*. *cenocepacia* K56-2 and *B*. *stabilis* LMG12494. Knockdown of gp91 resulted in a slight increase in bacterial burden compared to infected control embryos, although this was only significant for *B*. *stabilis* (Fig 7A, 7B and 7C). In addition, neutrophils were still massively recruited in 71.3% (±14.43% SEM) of the gp91 knockdown embryos infected with *B*. *stabilis* at 21 hpi (n = 8, 10, and 22 respectively, and Fig 7C) compared to 13.2% (as indicated above) for control embryos, showing that the absence of Gp91 reduces the host’s ability to resolve inflammation elicited by this strain.

Together the data show that even upon neutrophil-dominated phagocytosis, virulent *B*. *cenocepacia* K56-2 exerts its pro-inflammatory effects through macrophages. A functional ROS response helps the host to slow down multiplication of both *B*. *cenocepacia* and *B*. *stabilis* to some extent, and contributed to the resolution of inflammation during infection with *B*. *stabilis*, although no effect was seen with the virulent *B*. *cenocepacia*.

### Positive and negative effects of Il1-signalling for the *B*. *cenocepacia*-infected host

Studies in mice have implicated Myd88, a key adaptor protein in innate immune signalling, in the pathological inflammation associated with *B*. *cenocepacia* infection [31]. In zebrafish, we found that the absence of Myd88 resulted in reduced mortality, with *B*. *cenocepacia*-infected *myd88*^*-/-*^ mutant embryos living significantly longer than infected *myd88*^*+/+*^ embryos (S7A Fig), but no difference was observed in bacterial burden (S7B Fig). The reduced mortality found in *myd88*^*-/-*^ mutants, however, was not consistently mimicked by morpholino knockdown of myd88, although no significant increase in mortality was found compared to control embryos (S7C Fig and S7D Fig). The data show that Myd88 is not critical for host defence, and suggest that Myd88-dependent and -independent signalling pathways contribute to fatal pro-inflammatory disease. The lack of a consistent host-protective effect of *myd88* deficiency led us to focus on pro-inflammatory cytokines that might contribute to the fatal inflammation in *B*. *cenocepacia*-infected embryos.

**Fig 8:**
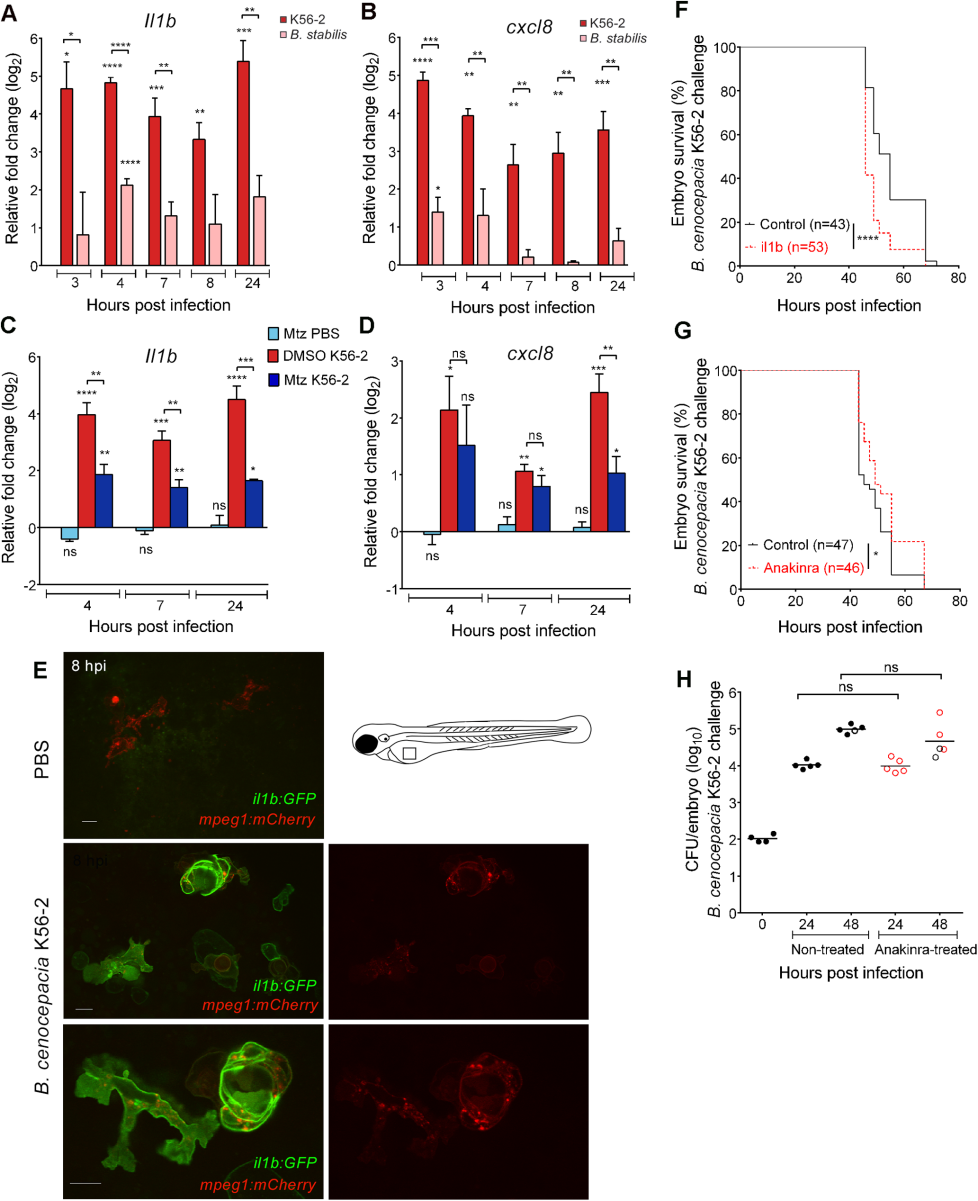
*B*. *cenocepacia* K56-2 induces robust pro-inflammatory *Il1b* expression that is dependent on macrophages. **(A,B)** Mean relative *il1b* **(A)** and *cxcl8* **(B)** gene expression levels (qRT-PCR) in embryos injected with on average 250 CFU *B*. *cenocepacia* K56-2 (red bars) or on average 111 CFU *B*. *stabilis* LMG14294 (pink bars), normalized to a PBS-injected control group at each time point. Error bars represent mean with SEM of three biological replicates. Asterisks above each bar indicate significance compared to the PBS control at each time point, significance between groups per time point is indicated with a horizontal line. **(C,D)** *mpeg1/*umn^*+*^ embryos were pre-treated at 34 hpf for 15 h with DMSO or 5 mM Mtz. Randomized groups were injected with either PBS or with *B*. *cenocepacia* K56-2 (on average 150 CFU). Graphs show mean relative *il1b* **(C)** and *cxcl8* **(D)** gene expression levels (qRT-PCR) normalized to the PBS-injected DMSO-treated group at each time point. Error bars represent mean with SEM of two biological replicates. See also S8 Fig. **(E)** Confocal stack images (green/red overlay (left panels) and red channel (right panels)) of *il1b*:*GFP*/*mpeg1*:*mCherry* embryos 8 h post iv injection with PBS, or *B*. *cenocepacia* K56-2 (DS-Red). Due to strong fluorescence of GFP in epithelial cells in the trunk and head region, images were taken over the yolk sac valley. Scale bars 10 μm. **(F)** Embryo survival (average inoculum 44 CFU, representative experiment) of control (black) and il1b knockdown embryos (red) injected iv with *B*. *cenocepacia* K56-2. **(G,H)** Embryo survival **(G)** and bacterial burden over time **(H)** of control (black) and Anakinra-treated embryos (red) injected iv with *B*. *cenocepacia* K56-2 (average inoculum 107 CFU for both groups). Representative experiment. **(A-D, F-H)** * p ≤ 0.05; ** p ≤ 0.01; *** p ≤ 0.001; **** p ≤ 0.0001; ns: non-significant.

To determine pro-inflammatory responses playing a role during acute and persistent infection of zebrafish embryos, we analysed global *cxcl8* and *il1b* gene expression levels. In wildtype embryos, *B*. *cenocepacia* K56-2 induced a rapid and robust increase in *il1b* and *cxcl8* expression, which remained high until at least 24 hpi (Fig 8A and 8B). In contrast, injection of *B*. *stabilis* resulted in significantly lower induction of expression of these pro-inflammatory cytokine genes, in agreement with the persistent character of the infection. To study the contribution of macrophages to the global increase in pro-inflammatory gene expression, we analysed infection-induced changes in *il1b* and *cxcl8* expression in embryos that were depleted of macrophages using Mtz-mediated cell ablation. Although no significant difference was found in infection-induced *cxcl8* expression at 4 and 7 hpi, the induction of *il1b* expression was significantly lower in *B*. *cenocepacia* K56-2 infected embryos that were depleted of macrophages compared to infected DMSO-treated control embryos (Fig 8C and 8D). Since *il1b* and *cxcl8* expression were not affected by Mtz or solvent (DMSO) treatment in K56-2 or PBS injected wildtype embryos (S8A Fig), nor in PBS-injected macrophage-depleted *mpeg1*/umn^+^ embryos (Fig 8C and 8D), these results show that zebrafish macrophages contribute significantly to the infection-induced increase in global *il1b* expression.

The involvement of macrophages in the induction of *il1b* expression was further confirmed using an *il1b-GFP* reporter fish line that expresses membrane-targeted GFP from the *il1b* promoter sequence [32]. Intravenous injection of *il1b*:*GFP*/*mpeg1*:*mCherry* embryos with *B*. *cenocepacia* K56-2 resulted in expression of GFP in macrophages within 2 hours, with strong expression from 4 hpi (Fig 8E), confirming that macrophages significantly contribute to the global increase in *il1b* expression levels.

We then knocked down the expression of *il1b*, which resulted in enhanced mortality (Fig 8F), suggesting that Il1b signalling contributes to host defence. However, treatment of larvae with Anakinra, an antagonist of the Il1 receptor, resulted in a small but significant increase in survival of *B*. *cenocepacia* K56-2 infected embryos (Fig 8G), although bacterial multiplication (Fig 8H) and infection-induced expression of *il1b* and *cxcl8* (S8B Fig), were not affected by Anakinra treatment. These apparently contrasting results suggest that Il1b contributes to host-protective and detrimental responses, while the balance of Il1-receptor mediated signalling leans towards fatal pro-inflammatory responses.

## Discussion

**Fig 9:**
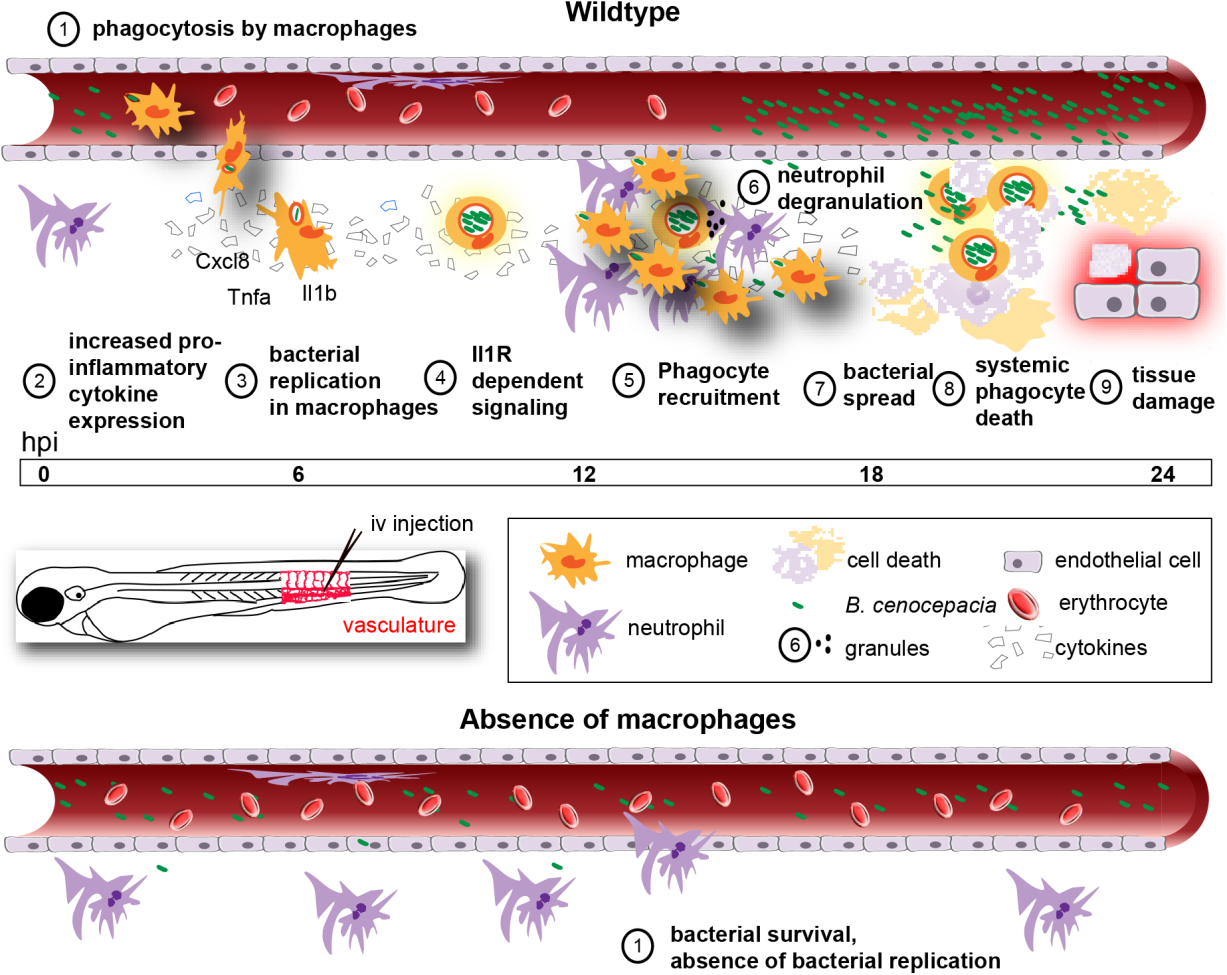
Schematic showing the role of macrophages during acute infection by *B*. *cenocepacia* K56-2 in zebrafish embryos. Macrophages are the major phagocytosing cells of iv injected bacteria (1) for which they provide a critical replication niche (3). In their absence, K56-2 does not replicate (bottom panel, 1). Intracellular bacteria induce a rapid and robust increase in pro-inflammatory cytokine expression (2). Il1 signalling contributes to fatal pro-inflammatory responses (4), characterized by massive neutrophil and macrophage infiltration (5) neutrophil degranulation (6), bacterial dissemination from infected cells (7), systemic phagocyte death (8), and tissue damage (9).

Macrophages have been suggested to be important for Bcc virulence and invasiveness in infected CF patients since it was shown for the first time in the late 90’s that Bcc bacteria can survive in macrophages *in vitro* [33]. Bcc bacteria are strong biofilm producers and it is generally assumed that, as seen with *Pseudomonas aeruginosa* [34], they exist and persist as biofilm communities in the lungs of infected CF patients. Recent clinical evidence has challenged this belief showing a strong association of Bcc bacteria with cells in lung tissue samples taken from transplant and deceased CF patients [35,36]. This was highlighted in the recent study by Schwab and colleagues who found *B*. *cenocepacia* mainly in macrophages and not in biofilm-like structures in CF lung tissue [36]. *In vitro*, CF and CGD macrophages are more permissive to infection with *B*. *cenocepacia* than their healthy counterparts; however, these bacteria have also been shown to replicate in macrophages derived from healthy individuals, sometimes even to high numbers as exemplified for human monocyte-derived macrophages [11,13]. Of note, *B*. *cenocepacia* has been detected in macrophages present in deep skin infections of dogs that were receiving cyclosporine [37]. Thus, macrophages may contribute to pathogenicity caused by this group of bacteria not only in CF, but also in nosocomial infections of both immune competent and immune suppressed individuals [2–4,38]; the contribution of (infected) macrophages to pathogenesis *in vivo* is, however, still unexplored. Here we provide *in vivo* evidence that zebrafish macrophages are critical for multiplication of *B*. *cenocepacia* in the host, and subsequent induction of pro-inflammatory fatal infection (Fig 9). A key role for intramacrophage stages in pathogenicity has important consequences for the design of novel antimicrobial therapies.

Many intracellular bacterial pathogens exploit macrophages for their virulence, however, the increased virulence of for instance *Mycobacterium marinum* [39], *Staphylococcus aureus* [40], and *Salmonella* Typhimurium [41] in macrophage-depleted zebrafish larvae shows that these phagocytes still have a beneficial role for the host. In sharp contrast, we show that genetic and chemical ablation of macrophages prior to infection considerably attenuated the virulence of Bcc strains that cause rapidly fatal infection in the macrophage-proficient host. Strikingly, in contrast to for instance *S*. Typhimurium [41], none of the analysed Bcc strains could replicate extracellularly in the absence of macrophages. This not only reveals the critical role of macrophages in proliferation of these bacteria and ensuing pro-inflammatory disease in the host, it also suggests that specific conditions prevented extracellular replication of the analysed strains upon introduction in the macrophage-depleted host. Of note, we have shown earlier that at later stages of infection *B*. *cenocepacia* re-enters the circulation and can replicate extracellularly [17], suggesting changes in host- or bacterial factors during intracellular or acute stages of infection support further extracellular bacterial proliferation. Our current research is focused on better understanding the inability of these strains to replicate extracellularly upon their delivery into the host, and whether this is a general feature of bacteria belonging to the Bcc.

Despite a significant reduction in bacterial multiplication in embryos experimentally depleted of macrophages, at later time points bacterial burden increased. This was more evident in pu.1 knockdown embryos than in embryos that were depleted of macrophages using Mtz-mediated cell ablation. It is not completely clear where *B*. *cenocepacia* starts to replicate at later time points in embryos depleted of macrophages but we believe that this is either in newly developed macrophages or, with Mtz, in macrophages that have survived treatment due to partial silencing of expression of the *mpeg*:*Gal4*/*uas*:*mCherry-NTR* transgenes. Further experiments are needed to determine whether persisting bacteria can enter and replicate in non-phagocytic cells in this model.

The pro-inflammatory state during *B*. *cenocepacia* infection was characterized by massive neutrophil and macrophage recruitment, neutrophil degranulation at the site of infection and tissue damage, culminating in fatal infection. The observed systemic phagocyte clearance might contribute to the pathological effects seen in zebrafish larvae. However, embryos that were experimentally depleted of neutrophils at the time of bacterial injection died as fast as infected neutrophil-proficient embryos. Thus the net contribution of neutrophils to disease outcome, whether host-protective or harmful, was undetectable, and emphasizes the pathological effect of the interaction between *B*. *cenocepacia* and macrophages.

In agreement with the finding that neutrophils need a surface for efficient phagocytosis [29], we show that neutrophils were massively recruited to and readily phagocytosed *B*. *cenocepacia* K56-2 and *B*. *stabilis* delivered by subcutaneous injection in zebrafish embryos, although macrophages were also seen to take up bacteria. As observed after iv delivery, *B*. *cenocepacia* caused pro-inflammatory disease with sustained infiltration of neutrophils and macrophages, and bacterial burden strongly increased, whereas *B*. *stabilis* was less virulent and most recruited neutrophils had left the infection site by 24 hpi. Experimental depletion of neutrophils prior to subcutaneous injection of *B*. *cenocepacia* did not have a major effect on bacterial replication and acute disease progression. In sharp contrast, the absence of macrophages prevented multiplication of *B*. *cenocepacia* and tissue damage. Thus, also with neutrophil-dominated phagocytosis and tissue localization of the bacteria, macrophages were critical for acute disease progression, as seen after intravenous delivery.

It has been reported that neutrophils which are unable to produce ROS through defects in the NADPH oxidase complex, as is the case in CGD patients, showed reduced killing of ingested Bcc, with the efficiency of killing depending on the bacterial strain [8]. CGD neutrophils were also unable to protect mice against Bcc challenge [42,43]. We did not find a significant effect of gp91 knockdown on virulence prior to intravenous injection of *B*. *cenocepacia* or *B*. *stabilis*, in agreement with our finding that neutrophils do not contribute measurably to disease outcome. However, subcutaneous delivery revealed that *B*. *stabilis* numbers increased slightly more in gp91 knockdown embryos compared to controls, indicating that in zebrafish embryos ROS plays some restrictive role against this strain. For *B*. *cenocepacia*, bacterial numbers in gp91 knockdown embryos were not significantly increased compared to those in control embryos, although we cannot exclude at this point that the dominant role of macrophages in virulence of *B*. *cenocepacia* masked a ROS-dependent effect.

While subcutaneously delivered *B*. *cenocepacia* showed sustained neutrophil recruitment at later time points even in the presence of functional NADPH oxidase, in most *B*. *stabilis*-infected embryos recruited neutrophils had left the infection site at later time points in a ROS-dependent manner, suggesting ROS play a role in the resolution of inflammation. The inability to efficiently resolve inflammation is reminiscent of neutrophil dominated abscesses found in the lungs of rats lacking ROS [43], which was contributed to Bcc-induced necrosis of neutrophils lacking a functional NADPH oxidase [44]. Starting around 1 hpi, neutrophils that had taken up *B*. *stabilis* or *B*. *cenocepacia* rounded up and expelled their cell contents into the environment as described for NETosis [45]. NETosis, a more recently described immune defence mechanism, depends on the production of ROS by NADPH oxidase [46]. Our results suggest that ROS-dependent release of NETs might be a host-protective mechanism to control *B*. *stabilis* infection. Together, our data demonstrate that macrophages, and not neutrophils, critically contribute to the detrimental effects during acute infections caused by intravenously and subcutaneously introduced *B*. *cenocepacia*.

Detection of bacterial ligands through Toll-like receptors (TLR) is essential for initiation of downstream immune signalling, principally through the key adaptor protein MYD88. Flagellin has been suggested as a key ligand in TLR5-mediated induction of the immune response to infection with Bcc bacteria [47,48]. However, recently it has also been described that glycosylation of *B*. *cenocepacia* flagellin reduces immune responses [49], and it remains essential to identify other bacterial ligands and host factors that are involved in the induction of this high, deregulated immune response and the inability to resolve the inflammation. In contrast to other pathogens, including *Edwardsiella tarda* and *S*. Typhimurium that have been shown to be more virulent in a zebrafish *myd88*^*-/-*^ mutant [41], we found that *B*. *cenocepacia*-infected *myd88*^*-/-*^ mutants lived longer than wildtype embryos. Although these findings are in agreement with results of Ventura et al [31] who showed that immunocompromised mice lacking MyD88 were protected against fatal challenge with *B*. *cenocepacia*, we were unable to reproducibly mimic these findings in myd88 knockdown experiments. Despite this inconsistency, however, the increased virulence in the absence of Myd88 described for other pathogens [41] was not observed, showing Myd88 does not have a measurable host-protective role against Bcc. Differences in experimental conditions and fish lines may contribute a difference in the balance between Myd88-dependent and -independent pathways, making it impossible to unmask a host detrimental role for Myd88 under certain conditions. Alternatively, Myd88-dependent host-protective and host detrimental effects may cancel each other out, and further research is needed to clearly define a role for Myd88 in acute fatal infection caused by *B*. *cenocepacia* in zebrafish.

Secretion of the pro-inflammatory cytokine IL1β has been shown to be strongly induced upon infection of macrophages and epithelial cells with *B*. *cenocepacia*. Activation of the pyrin inflammasome in monocytes and THP-1 cells by intracellular *B*. *cenocepacia* has been shown to be important for processing and secretion of IL1β in a type VI-secretion dependent manner [20]. We found that intracellular *B*. *cenocepacia* caused a rapid and robust increase in pro-inflammatory cytokine gene expression in zebrafish embryos, which contrasted to the low increase induced by *B*. *stabilis*, in agreement with the acute and persistent character of the infection, respectively. During acute infection, the induction of *il1b*, but not *cxcl8* expression, was significantly lower in the absence of macrophages indicating an important role for macrophages in the global infection-induced *il1b* expression in zebrafish embryos. Knockdown of il1b enhanced host mortality, showing that Il1b is important for host defence against *B*. *cenocepacia*. However, treatment with the IL-1 receptor antagonist Anakinra, which has been approved in humans for the treatment of pro-inflammatory effects of rheumatoid arthritis, resulted in increased resistance of zebrafish larvae towards *B*. *cenocepacia*. Our results suggest that while Il1b contributes to pro-inflammatory responses that can either be host-protective or detrimental to the host, its absence is disadvantageous. The finding that Il1R signalling, which generally has a protective role in host defence, is detrimental for embryos infected with *B*. *cenocepacia*, was also reported in a mouse model of melioidosis, where IL-1r1^-/-^ mice were more resistant than wildtype mice to *B*. *pseudomallei* infection [50]. A possible mechanism for the enhanced resistance to *B*. *cenocepacia* K56-2 by antagonising the Il1R receptor could be through a role for Anakinra in modulation of autophagy [51]. *B*. *cenocepacia* has been shown to reduce the expression of autophagy related genes [12] and to block autophagosome completion [13]. We are currently investigating a possible role for Anakinra in the restoration of autophagy in zebrafish macrophages infected with *B*. *cenocepacia*.

To date the molecular mechanisms that are at the basis of exacerbation, decline of lung function and often fatal septicemia in CF patients caused by Bcc bacteria are not known. Equally, it is unknown how these opportunists cause disease in both immune compromised and immune competent individuals. Based on our results, and those from others, we suggest that the intracellular stages of *B*. *cenocepacia* and the ensuing inflammatory response are essential therapeutic targets for the development of new therapies to combat these infections. We propose that zebrafish embryos are a valuable additional model to help advance our understanding of the interaction of Bcc bacteria with macrophages and neutrophils in CF and non-CF conditions in the context of an innate immune system. The importance of our findings is highlighted by the recent findings that several important extracellular opportunistic pathogens, including *Staphylococcus aureus* and *Pseudomonas aeruginosa* which cause the large majority of nosocomial infections, have only recently been reported to be able to survive, and replicate in host cells [52–54]. Thus, increased knowledge on the precise infection mechanisms of such opportunistic pathogens is crucial for the design of more effective antimicrobial therapies to disarm intracellular bacteria.

## Materials and methods

### Ethics statement

Zebrafish (*Danio rerio*) were kept and handled in compliance with the guidelines of the European Union for handling laboratory animals (http://ec.europa.eu/environment/chemicals/lab_animals/home_en.htm). Studies performed at VBMI are approved by the Direction Départementale de la Protection des Populations (DDPP) du Gard (ID 30-189-4) and the Comité d'Ethique pour l'Expérimentation Animale Languedoc-Roussillon (CEEA-LR-12186). Culture of *myd88* mutant zebrafish was approved by the local animal welfare committee (DIerexperimentencommissie, DEC) of the University of Leiden (protocol 12232) and adhered to the 2010/63/EU directive. Infection-experiments were terminated before the larvae reached the free feeding stage and did not classify as animal experiments according to the 2010/63/EU Directive.

#### Zebrafish

Care and maintenance of zebrafish was as described previously [17]. Zebrafish lines used in this study are summarized in Table 1.

**Table 1:** Zebrafish lines used in this study.

Name	Description	Reference
**AB**	Wildtype	-
**AB “Golden”**	Pigment mutation (*slc24a5*)	[55]
***Tg*(*mpx*:*eGFP*^*i114*^)**	Neutrophil marker–GFP, referred to as *mpx*:*GFP*	[56]
***Tg*(*mpeg1*:*mCherryF*) ^ump2Tg^**	Macrophage marker with membrane localised mCherry-F, referred to as *mpeg1*:*mCherry*	[32]
***Tg(fms*:*Gal4-VP16*^il86/^*/UAS-E1b*:*nfsB-mCherry*^*i149*^)**	Fish line expressing a fusion protein of nitroreductase and mCherry under control of the Gal4 regulated UAS sequence in macrophages	[57]
***Tg*(*mpeg1*:*Gal4-VP16/ UAS-E1b*:*kaede*^*s1999t*^)**	Line expressing the photo convertible protein Kaede under control of the Gal4-regulated UAS sequence in macrophages	[58]
***myd88*^hu3568^**	Mutant line with truncated MyD88, referred to as *myd88*^*-/-*^	[41]
***Tg*(*mpeg1*:*Gal4-VP16-*^il86/^; *UAS-E1b*:*nfsB-mCherry*^*i149*^)**	Offspring from a cross between Tg(*fms*:*Gal4;* *UAS-E1b*:*nfsB-mCherry*^*i149*^) and Tg(*mpeg1*:*Gal4-VP16* */UAS-E1b*:*kaede*^*s1999t*^) fish, referred to as *mpeg1*/umn	[57, 58, this study]
***Tg*(*mpx*:*Gal4-VP16/ UAS-E1b*:*nfsB-mCherry*^*i149*^)**	Offspring selected from a cross between Tg(*mpx*:*Gal4*.*VP16*)^i222^;Tg(*UAS*:*Kaede*)^s1999t^ (59) and Tg(*mpeg1*:*Gal4-VP16-* ^il86/^; *UAS-E1b*:*nfsB-mCherry*^*i149*^), referred to as *mpx*/umn	[57–59, this study]
***Tg*(*il1b*:*GFP*)**	Fish expressing farnesylated GFP (membrane-targeted GFP; GFP-F) under the control of the *il1b* promoter, named *il1b*:*GFP*	[32]

### Bacterial strains and plasmids

Bcc strains used in this study are: *B*. *cenocepacia* K56-2/LMG18836 [60], *B*. *cenocepacia* J2315/LMG16656 and J415/LMG16654 [61], *B*. *stabilis*/LMG14294 [62], *B*. *vietnamiensis* FC441/LMG18836 [63], and *B*. *cepacia* CEP509/LMG18821 [63]. The *mTurquoise2* gene (referred to as Turquoise) was amplified from pmTurquoise2-C1 [64] using primers TurquoiseNdeI-Fw (5’–GGAATTCCATATGGTGAGCAAGGGCGAGGAGC-3’; *NdeI* underlined) and TurquoiseXbaI-Rv (5’–GCTCTAGACTACTTGTACAGCTCGTCCATGCCGAG-3’; *XbaI* underlined), and cloned as an *NdeI*/*XbaI* fragment in pIN29 [17], replacing the DSRed gene, resulting in pIN289. Similarly, the eGFP gene was amplified from pEGFP-C3 (CLONTECH) using primers peGFP-Nde (5’–GGAATTCCATATGGTGAGCAAGGGCGAGGAG-3’; *NdeI* underlined) and peGFP-Xba (5’–GCTCTAGAATCACTTGTACAGCTCGTCCATGCCG-3’; *XbaI* underlined) and cloned as *NdeI*/*XbaI* fragment in pIN29, resulting in pIN301. The plasmids were transferred to *B*. *cenocepacia* by electroporation as described [17], and fluorescence verified. Bcc strains containing DsRed (pIN29 [17],Turquoise (pIN289, this study), or eGFP (pIN301, this study)) expression plasmids were grown in 5 ml of LB medium in the presence of 100 μg.ml^-1^ chloramphenicol at 37°C overnight.

### Zebrafish lines and injection conditions

One to three nL of morpholinos (MO) (Genetools, Eugene, OR) dissolved in H_2_O with 0.05% Phenol Red (see S1 Table) was micro injected in the yolk at the 1–2 cell stage and eggs were incubated at 28°C in E3 medium (5mM NaCl, 0.17mM KCl, 0.33mM CaCl2, 0.33 mM MgSO4, 0.5x10^-4^% methylene blue). Methylene Blue was omitted when embryos were used for pixel count analysis to avoid strong autofluorescence of the yolk. Just prior to injection of bacteria the dechorionated embryos were anesthetized in 0.04% buffered 3-aminobenzoic acid ethyl ester methanesulfonate (tricaine, MS222) in E3. Embryos were micro injected in the blood island or caudal vein (30 hpf, 50 hpf), or subcutaneously (50 hpf). In knockdown experiments, no differences in virulence were found between embryos injected with nonspecific control MO compared to non-treated embryos (S1E Fig). MO-injected embryos were further compared to non-treated embryos (Control embryos). Embryos were manually dechorionated 2 hours before injection with Bcc bacteria. To prepare the inoculum, bacteria expressing fluorescent proteins were grown overnight in LB medium. Bacteria were collected by centrifugation at 3,000 g for 3 min and resuspended in PBS (Gibco) at the desired concentration with 0.05% phenol red solution (Sigma) for visualization of the injection. Subcutaneous injection was performed as described [29] with 0.1–0.5 nL of bacterial suspensions at an OD of 5. Iv injection was performed with 1–2 nL of bacterial suspension (50 CFU for survival assays and CFU counts, or 200 CFU for qRT PCR experiments) as described [17,65]. Embryos in each group were randomly attributed to survival assays after injection (exact numbers are indicated in survival graphs), or to CFU counts (n = 5 per experiment per indicated time point). Precise inoculum (T = 0) and bacterial burden, expressed as colony forming units (CFU), were determined by plating individually lysed embryos as described [65]. Briefly, after injection, embryos were rinsed in E3 medium, anesthetized in tricaine, and individually transferred to 1.5 ml Eppendorf tubes containing 45μl of 1x Trypsin-EDTA. Embryos were disrupted by pipetting (20–30 times) and 50 μL 2% Triton X-100 was added, mixed by flicking the tube, and incubated for 30 min at room temperature. To complete disruption the mixture was pipetted up and down 20–30 times. Depending on the number of bacteria, total lysate was plated on LB-agar plates with 100μg.ml^-1^ chloramphenicol or serial dilutions were made and 10μl of each dilution was deposited on a square LB-agar plate. Plates were incubated overnight at 37°C. The next day the number of colonies was determined. Graphs represent the individual number of CFU per embryo at the different time points. For treatment of embryos with Anakinra (Kineret), dechorionated embryos were incubated in 10 or 100 μM Anakinra in E3 for 4 hours prior to injection with bacteria. After injection, the treatment was continued with 10 μM Anakinra, and the medium was refreshed at 24 hpi.

### Macrophage and neutrophil ablation

Offspring from a cross between Tg(*fms*:*Gal4; UAS-E1b*:*nfsB-mCherry*^*i149*^) and Tg(*mpeg1*:*Gal4-VP16/UAS-E1b*:*kaede*^*s1999t*^) fish (see Table 1) was selected for strong expression of mCherry in macrophages (*mpeg*/umn^+^), and raised to adulthood. Similarly, offspring was selected from a cross between *Tg(mpx*:*Gal4*.*VP16)i222;Tg(UAS*:*Kaede)s1999t* and *Tg(mpeg1*:*Gal4-VP16- il86/; UAS-E1b*:*nfsB-mCherryi149)*, that specifically expressed mCherry in neutrophils, referred to as *mpx*/umn. Umn^+^ fish were crossed to AB, or heterozygote siblings were incrossed. For efficient ablation of macrophages and neutrophils, *mpeg1*/umn^+^ and *mpx*/umn^+^ embryos were preselected at 30 hpf for strong mCherry expression, and embryos with only few mCherry-positive cells were discarded. Non-fluorescent embryos from the same crosses were used as umn-negative (unm^-^) control. Metronidazole (Mtz; Sigma), freshly prepared in 0.2% DMSO, was used at 5 mM in E3. Fluorescent (umn^+^) and non-fluorescent **(**umn^-^) embryos were dechorionated and either left untreated (infection control), or incubated in 0.2%DMSO (DMSO effect) or 5mM Mtz (ablation and non-specific Mtz control, respectively) at 33 hpf for 15 h prior to iv injection of bacteria. The schedule for Mtz treatment and injection of bacterial suspensions in *mpeg1*/umn larvae, including controls, is shown in S3 Fig. Embryos were incubated at 28°C in the dark. Ablation was verified by fluorescence microscopy. Treatment of 15 h prior to infection, followed by an addition 24 h in Mtz, resulted in specific ablation of macrophages by the time of injection (see results). Mtz had no significant effect on log-phase growth of the bacteria in LB medium, and CFU counts were identical between DMSO and Mtz treatments. For complete ablation of neutrophils, 10 mM Mtz was used for pre-treatment. After injection 5 mM was applied; no difference in virulence was observed between pre-treatment with 5 or 10 mM.

### Analysis of gene expression by qRT-PCR

At the indicated time points, 10 to 25 embryos were processed for RNA isolation, cDNA synthesis, and qRT-PCR analysis. The peptidylprolyl isomerase A-like (*ppial*) gene was used as a reference gene. For each condition, 10 to 25 embryos were transferred to 500μl of TRIzol, homogenized and stored at -80°C, as described [65]. RNA for each pool was extracted as described [66] and purified using the RNeasy MinElute Cleanup kit (Bio-Rad). Reverse transcription of each sample (500ng total RNA) was performed with the iScript cDNA synthesis kit (Bio-Rad) according to the manufacture. Quantitative RT-PCR was performed using the LightCycler 480 SYBR Green I Master mix (Roche), on a LightCycler 480. Each reaction was performed in a 10μl volume comprised of 2.5μl 10-fold diluted cDNA, 5μl of master mix and 10 pmol of each of the primers (S2 Table). Cycling parameters were: 95°C during 10 min to activate the polymerase, followed by 45 cycles of 95°C for 15 s and 60°C for 40 s. Fluorescence measurements were taken at the end of each cycle. Melting curve analysis was performed to verify that no primer dimers and non-specific product were amplified. Stability of the house keeping gene *ppial* was verified for each experiment. Results were analysed using the ΔΔCt method and represented as column bar graphs normalized to a PBS-injected control group at each time point, unless mentioned otherwise. Three biological control experiments were performed, each with two technical replicates, unless stated otherwise.

### Cell death assay and Sudan black staining

Embryos were injected with 50–100 CFU red fluorescent *B*. *cenocepacia* K56-2, and 20 h later with 1 nL of the live cell impermeable nucleic acid stain SYTOX Green (1mM in DMSO, Invitrogen). As control embryos were injected with 1 nL of DMSO. Embryos were analysed between 30 min and 6 hours after injection of the dye by fluorescence microscopy. Sudan black staining was performed as described [67].

### Antibody labelling

Zebrafish embryos were fixed in 4% Paraformaldehyde (PFA) in PBS with 0.4% Tween20 during 2 hours at room temperature or overnight at 4°C. Embryos were washed 4 times during 30 min with PBST (0.1% Tween 20 in PBS). Blocking was performed during 2 h at room temperature in 5% goat serum in PBST and embryos were incubated with 5% goat serum/PBST containing an antibody against the pan-leukocyte marker L-plastin (1:500 with gentle shaking at 4°C overnight [68]. Embryos were washed (3 x 30 min) with PBST at RT. Embryos were incubated with 2% goat serum/PBT containing secondary antibody anti-rabbit coupled with Alexa 350 (1:250) (Life Technologies). Embryos were rinsed twice with PBST and prepared for microscopy analysis.

### Microscopy and fluorescent pixel quantification

Embryos were transferred to glass-bottom dishes (MatTek Corp., Ashland, MA) in E3 (inverted microscope) or embedded in 0.5% E3-agarose (confocal) containing 0.02% MS222. A Leica DM IRB inverted microscope (bright-field, differential interference contrast (DIC), and fluorescence imaging) coupled to a Coolsnap fx camera (Roper Scientific) was used. A Nikon AZ100 equipped for bright-field and fluorescence imaging, coupled with Coolsnap HQ2 (Roper Scientific) using MetaVue software was used to record full size embryos. Confocal microscopy was performed with an Olympus FV10i and images and movies were processed with Fluoview software and Image J. Images were processed further using Adobe Photoshop, and time-lapse videos made with image J (see specific details in movie legends). To quantify phagocytic cells, the fluorescent pixel quantification method was used as described [69]. Graphs depict macrophage and neutrophil numbers. BioImageXD [70] was used to obtain the image in S6A Fig.

### Statistical analysis

Statistical analysis was performed using Prism 6 (GraphPad). Survival assays are represented in Kaplan-Meier graphs and analysed with a Log rank (Mantel-Cox) test. Representative experiments are shown with total numbers of embryos (n) indicated for each of the groups. In experiments determining CFU, and macrophage and neutrophil counts significance between multiple selected groups was determined using one-way Anova, with Sidak’s Multiple Comparisons test (the compared groups are indicated with significance level on each graph). The graphs show a summary of multiple biological replicates (see legends). CFU counts were log transformed and are presented in dot graphs showing the geometric mean per time point, with each embryo represented by a single dot. qRT-PCR data were log_2_-transformed, and significance of the log_2_-transformed data was analysed using one-way Anova, with Tukey’s Multiple Comparison Test at each time point by comparing treatments with each other and the PBS control (in case of multiple groups per time point). Columns indicate mean fold-change with SEM. Significance in relative fold-change is indicated for each treatment normalized to the corresponding PBS control with an asterisk above the column, or between two treatments with a connective line. Significance is indicated with: ns, non-significant, *, *p* ≤ 0.05; **, *p* ≤ 0.01; ***, *p* ≤ 0.001; ****, *p* ≤ 0.0001. For some experiments, representative images are shown of at least 2 independent experiments, with a total of at least 20 embryos (Figs 6C, 6D and 8E).

## Supporting information

S1 Fig Related to Figs 1 and 2. **Role of macrophages during infection with Bcc strains causing either persistent or acute infection. (A-D G-J)** Embryo survival **(A, C, G, and I)** and bacterial burden (total of 2 experiments) over time **(B, D, H, J)** of control (black) and pu.1 knockdown embryos (red) injected iv with *B*. *cenocepacia* J2315 (A,B), *B*. *cepacia* CEP509 (C,D), *B*. *cenocepacia* J415 (G,H), and *B*. *vietnamiensis* FC441 (I,J), respectively. **(E)** Representative experiment (of at least three) showing embryo survival of control embryos (n = 47), pu.1 knockdown (n = 54) and nonspecific control MO (n = 52) embryos injected with *B*. *cenocepacia* K56-2 (average 53 CFU). **(F)** Representative fluorescence image at 24 hpi showing neutrophils (green) in an *mpx*:*GFP* pu.1 knockdown embryo injected with *B*. *cenocepacia* J2315 (red) (~50 CFU). Inset shows corresponding bright field image. Scale bar, 100 μm. **(B, D, H, J)** Geometric mean with each data point representing an individual embryo. Dead embryos are indicated as black open circles. **(A-E and G-J)** * p ≤ 0.05; ** p ≤ 0.01; *** p ≤ 0.001; **** p ≤ 0.0001; ns: non-significant. See materials and methods for statistical tests used.
(TIF)

S2 Fig Related to Fig 1C and 1D. **Pu.1 knockdown prevents *B*. *cenocepacia* K56-2 multiplication**. Fluorescence overlay images (red and green filters) of the indicated area (boxed area in embryo drawing) of two *mpeg1*:*mCherry* control embryos (left) and two *mpeg1*:*mCherry* pu.1 knockdown (right) imaged at 1, 3, 16, 20, 24, and 41 h after injection with ~50 CFU *B*. *cenocepacia* K56-2 harbouring pIN301 (green). The absence of macrophages prevents efficient bacterial replication. Bacteria in control embryos colocalise with macrophages (arrow heads). Fluorescent macrophages disappear from control embryos (>20 hpi). Appearance of macrophages in pu.1 knockdown embryos (white arrows).
(TIF)

S3 Fig Related to Fig 1. **Chemical ablation of macrophages using the NTR/Mtz system. (A)** Schematic representation and treatment schedule of the chemical ablation strategy based on the nitroreductase (NTR)/ metronidazole (Mtz) system, shown for macrophage-specific ablation. See Materials and methods for details. **(B)** Representative fluorescence images of non-treated and Mtz-treated *mpeg1*/umn^+^ embryos, showing the efficacy of the Mtz treatment. Residual red fluorescence in treated embryos represented apoptotic cells. Scale bar, 0.5 mm. **(C)** Quantification of macrophage numbers in *mpeg1*/umn^+^ embryos (untreated or treated at 34 hpf with 5mM Mtz) at 0, 15 and 39 hours after treatment as indicated in **(A)**. The efficacy of macrophage ablation by Mtz treatment was evaluated by pixel counting. The average macrophage numbers of two independent experiments (n = 6) are shown. ns, non-significant, ** *p* ≤ 0.01, **** *p* ≤ 0.0001. See material and methods for statistical analysis used.
(TIF)

S4 Fig Related to Fig 1E and 1F. **Mtz-mediated ablation of macrophages prevents *B*. *cenocepacia* K56-2 multiplication**. Fluorescence overlay images (red and green filter) of two DMSO-treated (left panels) and two Mtz-treated (right panels) *mpeg1/umn*^*+*^ embryos imaged at 1, 3, 24, and 48 h after iv injection with ~50 CFU *B*. *cenocepacia* K56-2 harbouring pIN301 (green). Bacterial replication in control embryos resulted in macrophage cell death, seen as mCherry positive debris (>24 hpi) (see also S5F Fig). At 28 and 48 hpi individual fluorescence images (green filter) are shown below the overlay images. Drawing indicates imaged area, marking the embryo sac extension (green) prone to autofluorescence. Arrow head indicates mCherry positive macrophages in apoptosis.
(TIF)

S5 Fig Related to Fig 3. **Behaviour and fate of neutrophils and macrophages during acute and persistent infection. (A).** Confocal stack images of a time series, first image ~20 hours after infection of an *mpx*:*GFP* embryo *with B*. *cenocepacia K56-2*. Patrolling neutrophil inspects heavily infected macrophage (white arrow). Individual bacteria (arrow head), possibly released from infected cell nearby (open arrow), are being moved around by the neutrophil. The last image shows the same area 90 minutes later with the infected macrophage still intact. **(B,C**) *Mpx*:*GFP* embryos (50 hpf) were injected with *B*. *stabilis* LMG14294. **(B)** Representative images of control and *B*. *stabilis* infected *mpx*:*GFP* embryos with increased neutrophil numbers. Neutrophils were more dispersed in *B*. *stabilis*-infected compared to non-infected control embryos, where most neutrophils were resting in the caudal hematopoietic tissue. **(C)** The number of neutrophils in infected and non-infected control embryos was determined by pixel counting at different time points after injection. Each data point represents one embryo. The graph represents one of the three experiments represented in Fig 3C, but includes an additional time point at 5 days post infection, which was not determined in the other 2 experiments. A percentage of embryos injected with *B*. *stabilis* contained more neutrophils than control embryos (see also **(B)**). *, *p* ≤ 0.05. **(D)** *Mpeg1*:*mCherry* embryos were injected with *B*. *cenocepacia* K56-2 (~45 CFU) and the number of macrophages was evaluated by pixel counting at 0 and 24 hpi (30 and 54 hpf, respectively). The results are related to the corresponding experiment shown in Fig 3D. Each data point represents one embryo. Significance was determined using a one-way Anova with Sidak’s Multiple Comparisons test. **** *p* ≤ 0.0001. Two independent experiments (n = 5). **(E)** Image showing *B*.*cenocepacia* K56-2 (red) in an L-plastin labelled macrophage (blue) at 24 hpi. Scale bar, 50 μm. Inset, magnification red/ blue filters, scale bar 10 μm. **(F)** Images (bright field, green/red overlay, and detailed image with red/green filter), of a non-infected *mpeg1*/umn^+^ embryo (left panels) and *mpeg1*/umn^+^ embryo (right panels) iv infected with *B*. *cenocepacia* K56-2 (green, arrows) at 24 hpi. The images are similarly treated to enhance the red fluorescence to visualize the mCherry positive debris. The close up shows individual macrophages (left panel, red), and red fluorescent debris (arrow heads). Related to Fig 3F.
(TIF)

S6 Fig Related to Figs 6 and 7. **Macrophages, but not neutrophils, contribute to increased bacterial burden and pro-inflammatory responses towards subcutaneous *B*. *cenocepacia*. (A)** *mpeg/*umn^+^ embryo infected subcutaneously at 2 dpf with *B*. *cenocepacia* K56-2 (green). Confocal stack (59 slices, 1 μm) at 24 hpi showing bacteria in macrophages. A close up of the indicated area is shown in 3D, with volume rendering for GFP signal and surface rendering of mCherry signal without (middle panel) and with 50% surface transparency (right panel). Scale bar 50 μm. **(B)** Fluorescence images showing non-infected *mpx*/umn^+^ and *mpeg1*/umn^+^ control embryos at 3 dpf, the time point that resembles 24 h post subcutaneous injection in Fig 6. **(C)** Image overlay (bright field, red and blue filters) of the tail region of an *mpx/*umn^+^ embryo after subcutaneous injection with *B*. *cenocepacia* K56-2 expressing Turquoise, presenting tissue damage by 24 hpi. Scale bar, 100 μm. **(D)** Image overlay (bright field, red, blue and green filters) of the tail region of an Mtz-treated *mpeg1/*umn^+^; *mpx*:*GFP* embryo 24 h after subcutaneous injection with *B*. *cenocepacia* K56-2 expressing Turquoise. Scale bar, 100 μm.
(TIF)

S7 Fig Myd88 does not have a measurable host-protective role against *B*. *cenocepacia*. **(A)** Embryo survival (average inoculum *35* CFU, representative experiment) **(B)** and bacterial burden (average of 2 experiments) over time of wildtype control (*myd88*^+/+^) and mutant (*myd88*^-/-^) embryos iv injected with *B*. *cenocepacia* K56-2. **(C, D)** Embryo survival (average inoculum 39 **(C)** and 75 **(D)** CFU) of control (black) and myd88 knockdown embryos (red) iv injected with *B*. *cenocepacia* K56-2.
(TIF)

S8 Fig Related to Fig 8. **DMSO, Mtz and Anakinra do not affect *il1b* and *cxcl8* expression levels. (A)** *mpx*:*GFP* embryos were either non-treated or pre-treated at 34 hpf for 15 h with DMSO or 5 mM Mtz. Randomized groups were injected with PBS or *B*. *cenocepacia* K56-2 (on average 270 CFU). Graphs show mean relative *il1b* and *cxcl8* gene expression levels, normalized to the PBS-injected non-treated control group at 4 hpi. Error bars represent mean with SEM of two biological replicates. **(B**) Control and Anakinra-treated *mpx*:*GFP* embryos were iv injected with PBS or *B*. *cenocepacia* K56-2 (average 120 CFU). Graphs show mean relative *il1b* and *cxcl8* gene expression levels, normalized to the PBS-injected non-treated control group at each time point. Error bars represent mean with SEM of two biological replicates. ns = non-significant.
(TIF)

S1 Table Related to experimental procedures. Morpholinos used in this study.
(DOCX)

S2 Table Related to experimental procedures. Primers used for qRT-PCR experiments.
(DOCX)

S1 Movie Related to Fig 5. **Phagocytosis of surface-associated *B*. *cenocepacia* by neutrophils.** Live imaging of an *mpx*:*GFP* embryo, injected subcutaneously at 50 hpf with DSRed expressing *B*. *cenocepacia*, every 90 seconds from 20 mins pi, for the duration of 75 mins. A time lapse of 50 confocal images with maximal intensity projection is shown (19 steps x 2 μm) at a rate of 2 frames per sec. Bacteria are rapidly phagocytosed by neutrophils, and remain visible in individual small vacuoles. Bacteria are also phagocytosed by GFP-negative macrophages, seen as individual bacterial clusters that are not colocalising with GFP-positive neutrophils.
(AVI)

S2 Movie Related to Fig 5. **Phagocytosis of surface-associated *B*. *stabilis* by neutrophils induces cell rupture.** Live imaging of an *mpx*:*GFP* embryo, injected subcutaneously over a somite at 50 hpf with DSRed expressing *B*. *stabilis*, every 90 seconds from 10 minutes pi. Total duration 128 mins representing the period between 20 mpi and 148 mpi. A time lapse of 85 confocal images with standard deviation projection is shown (19 steps x 2 μm) at a speed of 2 frames per sec. Bacteria are rapidly phagocytosed by neutrophils, and are directed into large growing vacuoles (See Fig 5). Between 64–67 mpi two neutrophils full of bacteria round up (arrows) and eject their cell contents into the extracellular space (arrow heads appear), see also Fig 5. After the event, the bacteria remain clustered, and mobility of surrounding neutrophils is dramatically reduced, before they start moving again. Other neutrophils (indicated with arrows and consecutive numbers 3, 4 etc) undergo the same phenomenon, although sometimes the collapse is less explosive. The neutrophil indicated with number 4 seems to expel part of its contents including the phagocytosed bacteria, but the neutrophil takes up the contents again and after 35 mins, effectively “bursts” (indicated with arrow 4).
(AVI)
