# Correction: Macrophages, but not neutrophils, are critical for proliferation of *Burkholderia cenocepacia* and ensuing host-damaging inflammation

**DOI:** 10.1371/journal.ppat.1006795

**Published:** 2017-12-20

**Authors:** Jennifer Mesureur, Joana R. Feliciano, Nelly Wagner, Margarida C. Gomes, Lili Zhang, Monica Blanco-Gonzalez, Michiel van der Vaart, David O’Callaghan, Annemarie H. Meijer, Annette C. Vergunst

Fig 4 is incorrect. In Panel B, mpeg1 should be mpx. The authors have provided a corrected version here.

**Fig 4:**
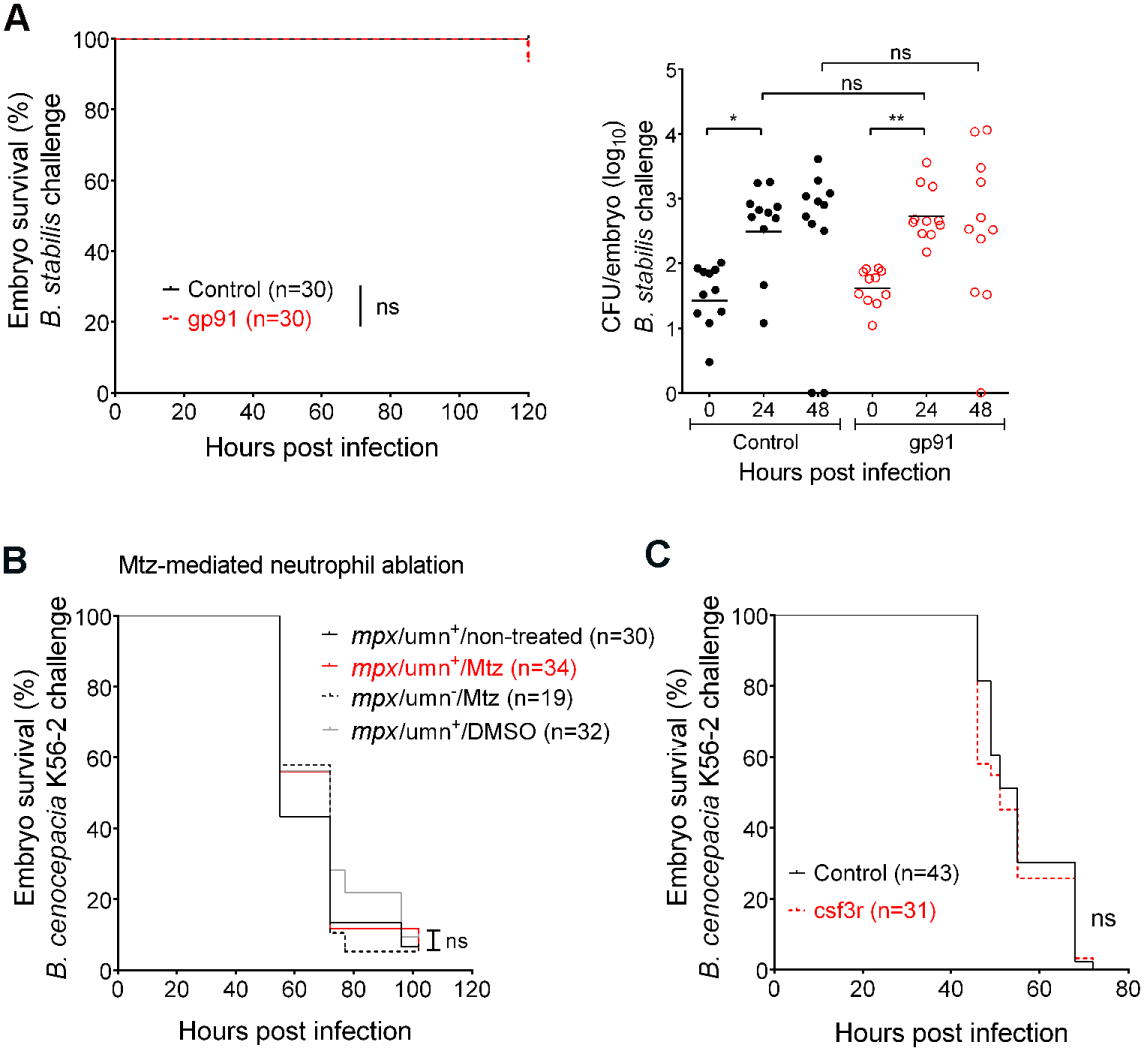
Neutrophils do not contribute significantly to infection. (A) Embryo survival (left; representative experiment) and bacterial burden (total of 2 experiments) over time (right panel, geometric mean) of control (black) and gp91 knockdown embryos (red) iv injected with B. stabilis. Each data point represents an individual embryo. (B) Embryo survival of mpx/umn+ embryos, untreated or treated with 10 mM Mtz or 0.2% DMSO, and mpx/umn−embryos treated with 10 mM Mtz iv injected with ~ 50 CFU B. cenocepacia K56-2. (C) Embryo survival (average inoculum 50 CFU, representative experiment) of control (black) and csf3R knockdown embryos (red) injected iv with B. cenocepacia K56-2. * p ≤ 0.05; ** p ≤ 0.01; ns: non-significant. See materials and methods for statistical tests.
