# Supplementary figures and images for: Macrophages, but not neutrophils, are critical for proliferation of Burkholderia cenocepacia and ensuing host-damaging inflammation
Source: PLoS Pathog. 2017 Jun 26;13(6):e1006437. doi: 10.1371/journal.ppat.1006437 (PMC5501683; doi:10.1371/journal.ppat.1006437)

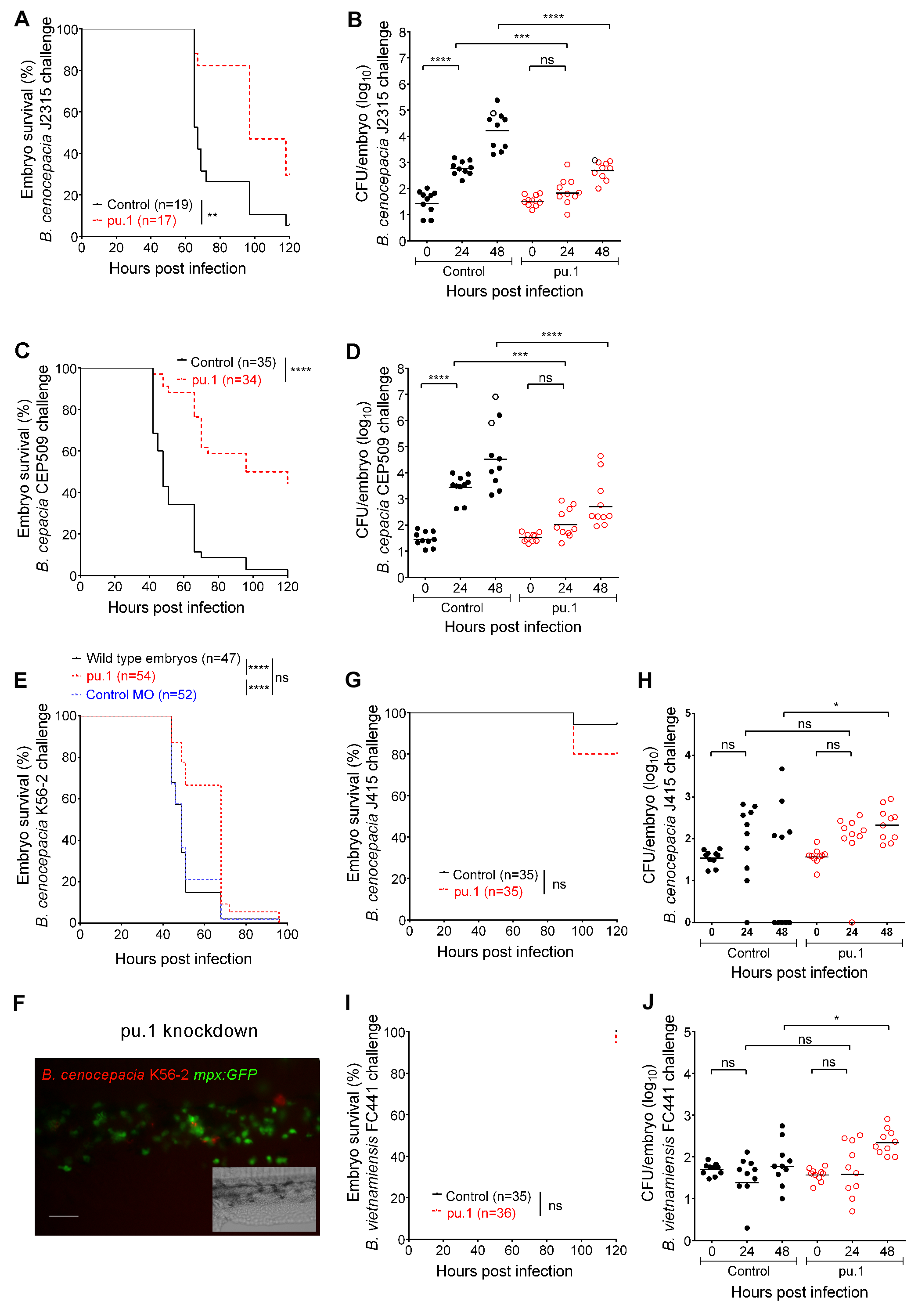

Supplement: S1 Fig — Role of macrophages during infection with Bcc strains causing either persistent or acute infection. (A-D G-J) Embryo survival (A, C, G, and I) and bacterial burden (total of 2 experiments) over time (B, D, H, J) of control (black) and pu.1 knockdown embryos (red) injected iv with B. cenocepacia J2315 (A,B), B. cepacia CEP509 (C,D), B. cenocepacia J415 (G,H), and B. vietnamiensis FC441 (I,J), respectively. (E) Representative experiment (of at least three) showing embryo survival of control embryos (n = 47), pu.1 knockdown (n = 54) and nonspecific control MO (n = 52) embryos injected with B. cenocepacia K56-2 (average 53 CFU). (F) Representative fluorescence image at 24 hpi showing neutrophils (green) in an mpx:GFP pu.1 knockdown embryo injected with B. cenocepacia J2315 (red) (~50 CFU). Inset shows corresponding bright field image. Scale bar, 100 μm. (B, D, H, J) Geometric mean with each data point representing an individual embryo. Dead embryos are indicated as black open circles. (A-E and G-J) * p ≤ 0.05; ** p ≤ 0.01; *** p ≤ 0.001; **** p ≤ 0.0001; ns: non-significant. See materials and methods for statistical tests used. (TIF) [file ppat.1006437.s001.tif]

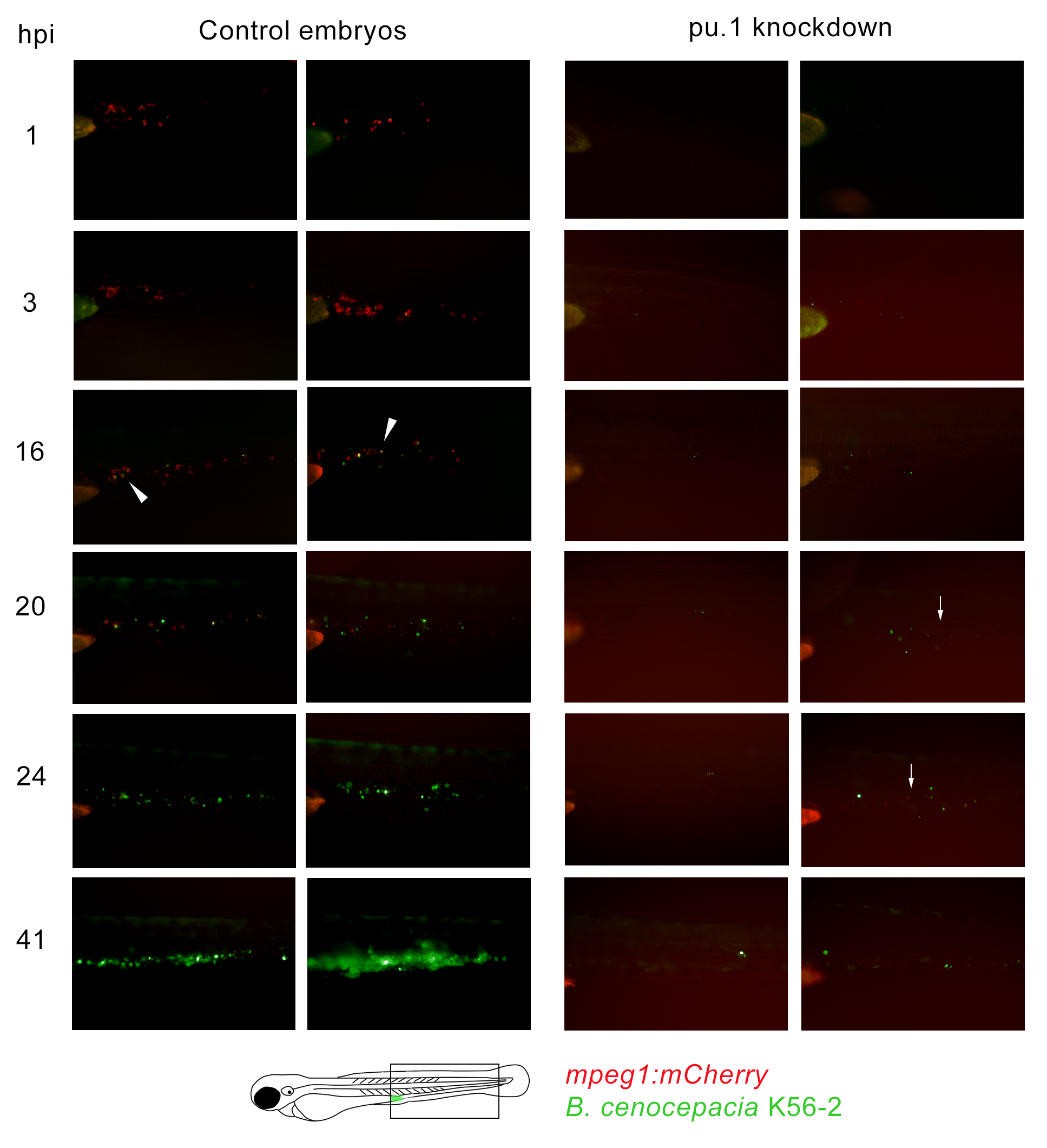

Supplement: S2 Fig — Pu.1 knockdown prevents B. cenocepacia K56-2 multiplication. Fluorescence overlay images (red and green filters) of the indicated area (boxed area in embryo drawing) of two mpeg1:mCherry control embryos (left) and two mpeg1:mCherry pu.1 knockdown (right) imaged at 1, 3, 16, 20, 24, and 41 h after injection with ~50 CFU B. cenocepacia K56-2 harbouring pIN301 (green). The absence of macrophages prevents efficient bacterial replication. Bacteria in control embryos colocalise with macrophages (arrow heads). Fluorescent macrophages disappear from control embryos (>20 hpi). Appearance of macrophages in pu.1 knockdown embryos (white arrows). (TIF) [file ppat.1006437.s002.tif]

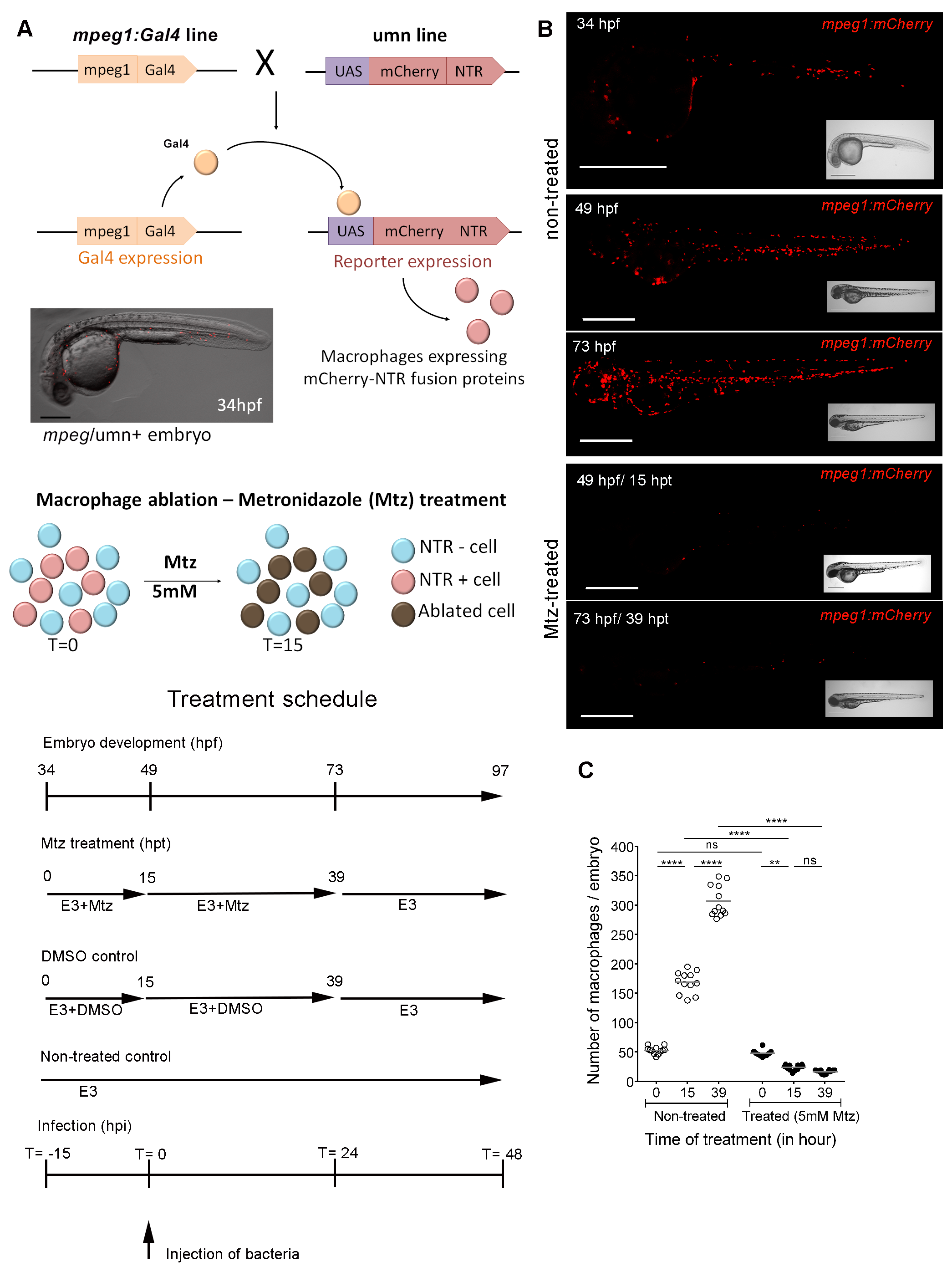

Supplement: S3 Fig — Chemical ablation of macrophages using the NTR/Mtz system. (A) Schematic representation and treatment schedule of the chemical ablation strategy based on the nitroreductase (NTR)/ metronidazole (Mtz) system, shown for macrophage-specific ablation. See Materials and methods for details. (B) Representative fluorescence images of non-treated and Mtz-treated mpeg1/umn+ embryos, showing the efficacy of the Mtz treatment. Residual red fluorescence in treated embryos represented apoptotic cells. Scale bar, 0.5 mm. (C) Quantification of macrophage numbers in mpeg1/umn+ embryos (untreated or treated at 34 hpf with 5mM Mtz) at 0, 15 and 39 hours after treatment as indicated in (A). The efficacy of macrophage ablation by Mtz treatment was evaluated by pixel counting. The average macrophage numbers of two independent experiments (n = 6) are shown. ns, non-significant, ** p ≤ 0.01, **** p ≤ 0.0001. See material and methods for statistical analysis used. (TIF) [file ppat.1006437.s003.tif]

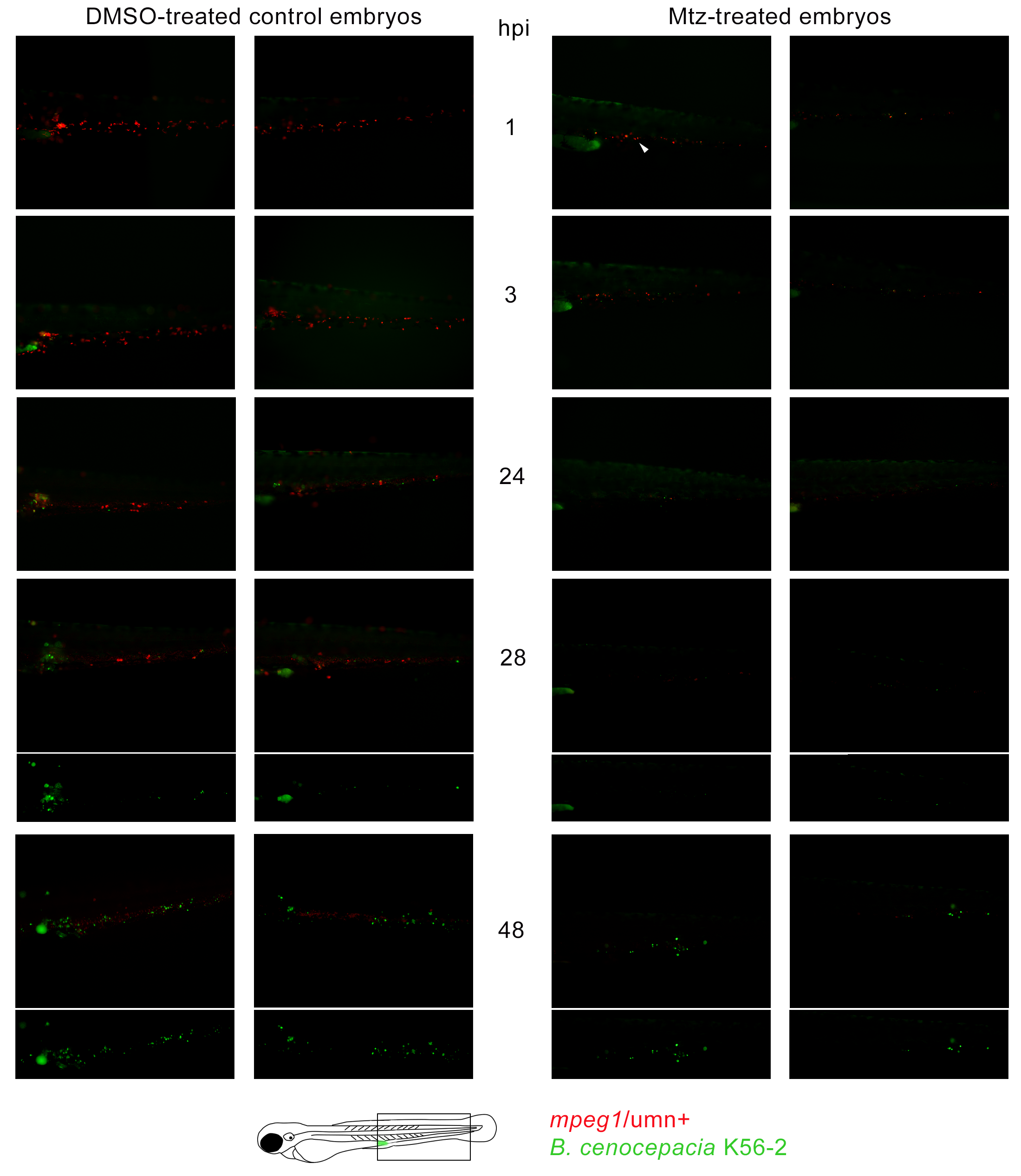

Supplement: S4 Fig — Mtz-mediated ablation of macrophages prevents B. cenocepacia K56-2 multiplication. Fluorescence overlay images (red and green filter) of two DMSO-treated (left panels) and two Mtz-treated (right panels) mpeg1/umn+ embryos imaged at 1, 3, 24, and 48 h after iv injection with ~50 CFU B. cenocepacia K56-2 harbouring pIN301 (green). Bacterial replication in control embryos resulted in macrophage cell death, seen as mCherry positive debris (>24 hpi) (see also S5F Fig). At 28 and 48 hpi individual fluorescence images (green filter) are shown below the overlay images. Drawing indicates imaged area, marking the embryo sac extension (green) prone to autofluorescence. Arrow head indicates mCherry positive macrophages in apoptosis. (TIF) [file ppat.1006437.s004.tif]

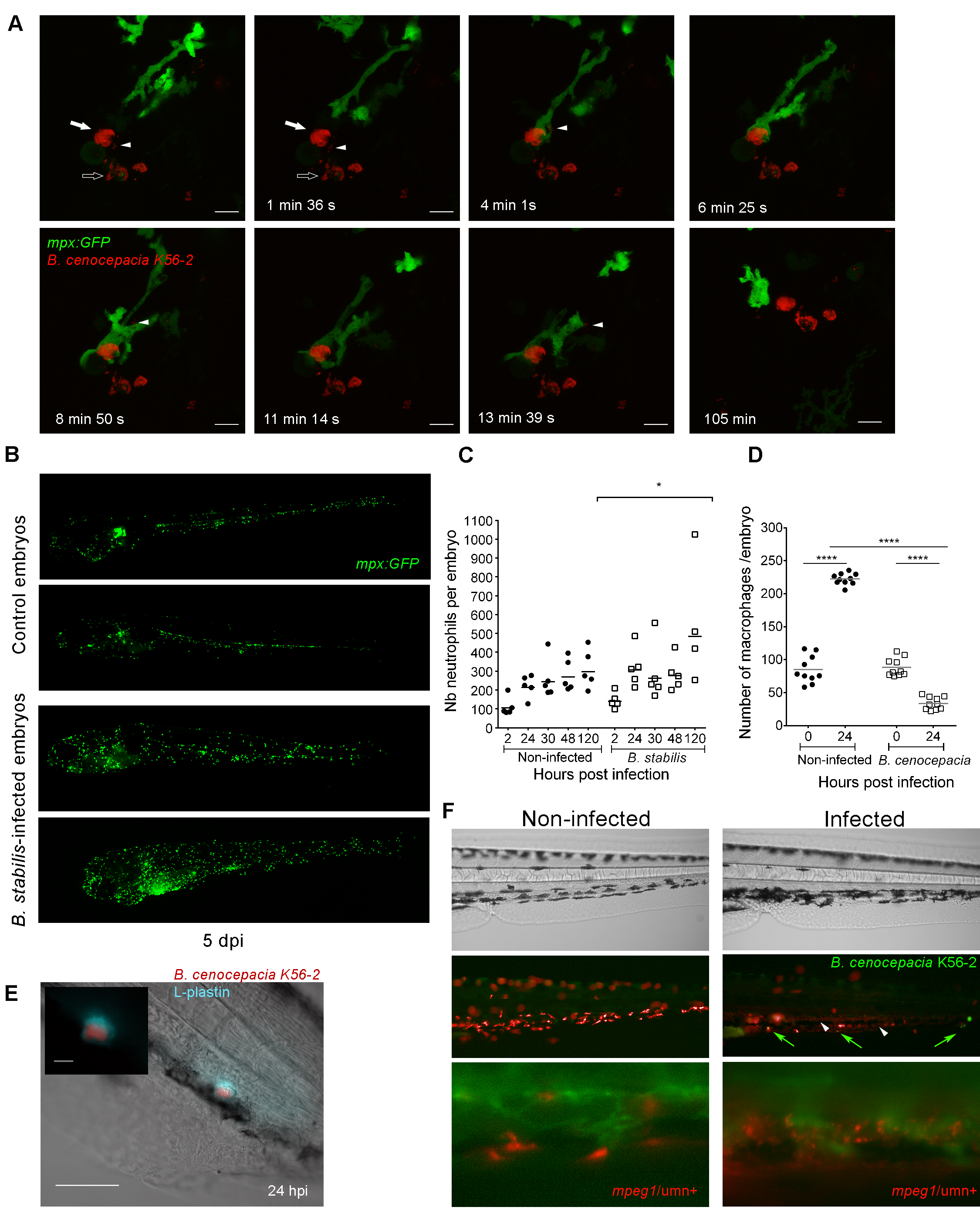

Supplement: S5 Fig — Behaviour and fate of neutrophils and macrophages during acute and persistent infection. (A). Confocal stack images of a time series, first image ~20 hours after infection of an mpx:GFP embryo with B. cenocepacia K56-2. Patrolling neutrophil inspects heavily infected macrophage (white arrow). Individual bacteria (arrow head), possibly released from infected cell nearby (open arrow), are being moved around by the neutrophil. The last image shows the same area 90 minutes later with the infected macrophage still intact. (B,C) Mpx:GFP embryos (50 hpf) were injected with B. stabilis LMG14294. (B) Representative images of control and B. stabilis infected mpx:GFP embryos with increased neutrophil numbers. Neutrophils were more dispersed in B. stabilis-infected compared to non-infected control embryos, where most neutrophils were resting in the caudal hematopoietic tissue. (C) The number of neutrophils in infected and non-infected control embryos was determined by pixel counting at different time points after injection. Each data point represents one embryo. The graph represents one of the three experiments represented in Fig 3C, but includes an additional time point at 5 days post infection, which was not determined in the other 2 experiments. A percentage of embryos injected with B. stabilis contained more neutrophils than control embryos (see also (B)). *, p ≤ 0.05. (D) Mpeg1:mCherry embryos were injected with B. cenocepacia K56-2 (~45 CFU) and the number of macrophages was evaluated by pixel counting at 0 and 24 hpi (30 and 54 hpf, respectively). The results are related to the corresponding experiment shown in Fig 3D. Each data point represents one embryo. Significance was determined using a one-way Anova with Sidak’s Multiple Comparisons test. **** p ≤ 0.0001. Two independent experiments (n = 5). (E) Image showing B.cenocepacia K56-2 (red) in an L-plastin labelled macrophage (blue) at 24 hpi. Scale bar, 50 μm. Inset, magnification red/ blue filters, scale bar 10 μm. (F [file ppat.1006437.s005.tif]

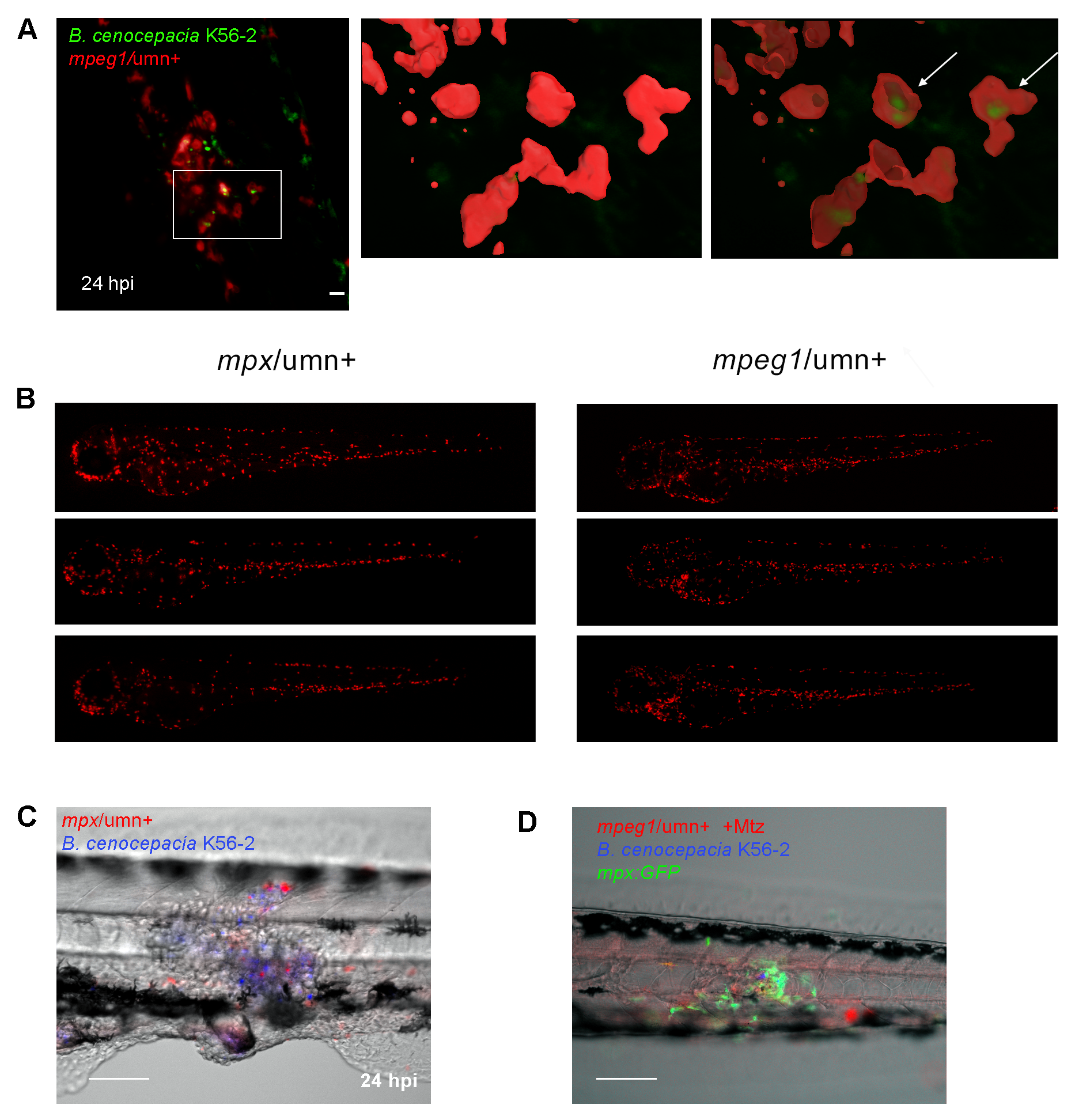

Supplement: S6 Fig — Macrophages, but not neutrophils, contribute to increased bacterial burden and pro-inflammatory responses towards subcutaneous B. cenocepacia. (A) mpeg/umn+ embryo infected subcutaneously at 2 dpf with B. cenocepacia K56-2 (green). Confocal stack (59 slices, 1 μm) at 24 hpi showing bacteria in macrophages. A close up of the indicated area is shown in 3D, with volume rendering for GFP signal and surface rendering of mCherry signal without (middle panel) and with 50% surface transparency (right panel). Scale bar 50 μm. (B) Fluorescence images showing non-infected mpx/umn+ and mpeg1/umn+ control embryos at 3 dpf, the time point that resembles 24 h post subcutaneous injection in Fig 6. (C) Image overlay (bright field, red and blue filters) of the tail region of an mpx/umn+ embryo after subcutaneous injection with B. cenocepacia K56-2 expressing Turquoise, presenting tissue damage by 24 hpi. Scale bar, 100 μm. (D) Image overlay (bright field, red, blue and green filters) of the tail region of an Mtz-treated mpeg1/umn+; mpx:GFP embryo 24 h after subcutaneous injection with B. cenocepacia K56-2 expressing Turquoise. Scale bar, 100 μm. (TIF) [file ppat.1006437.s006.tif]

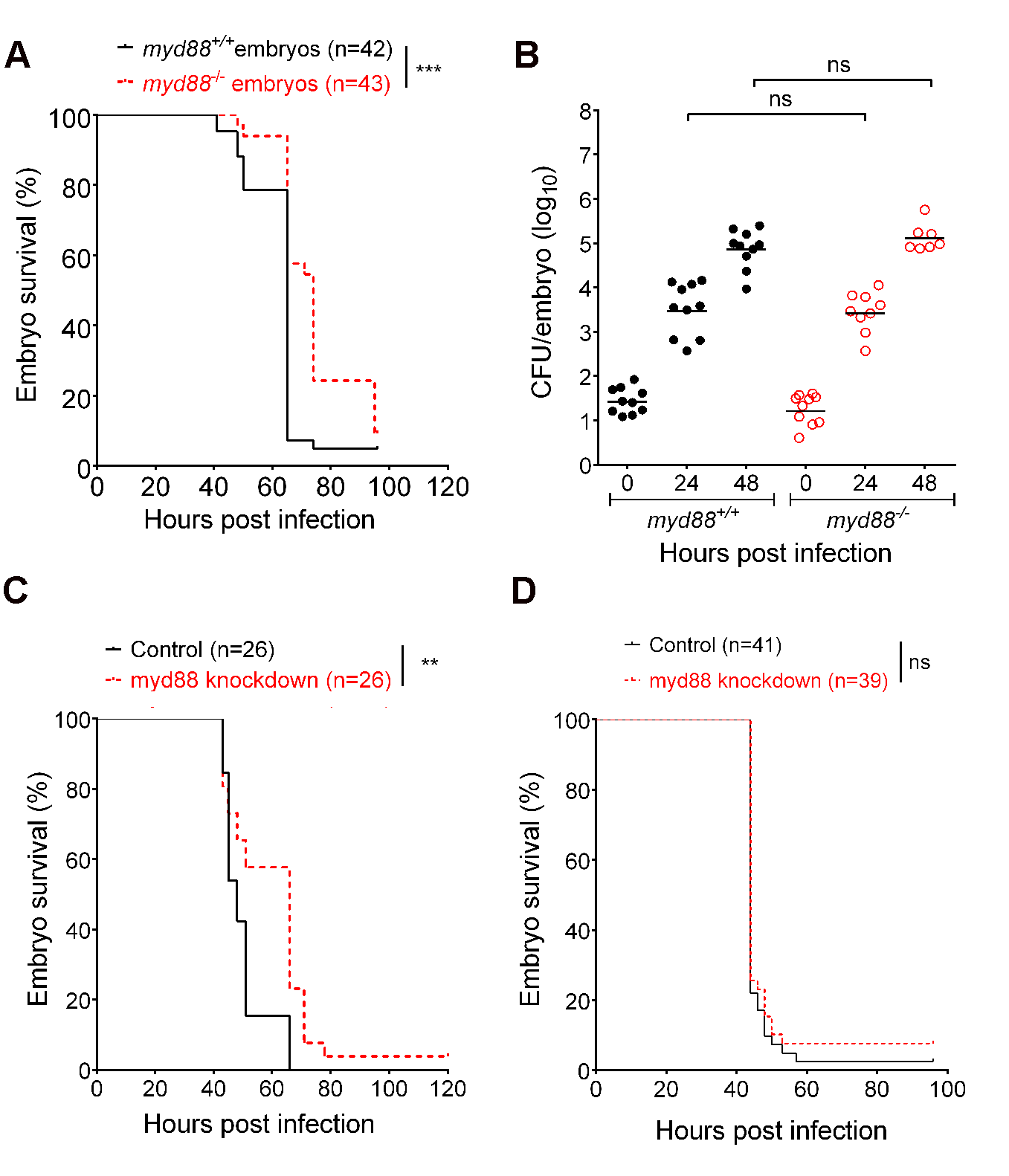

Supplement: S7 Fig — (A) Embryo survival (average inoculum 35 CFU, representative experiment) (B) and bacterial burden (average of 2 experiments) over time of wildtype control (myd88+/+) and mutant (myd88-/-) embryos iv injected with B. cenocepacia K56-2. (C, D) Embryo survival (average inoculum 39 (C) and 75 (D) CFU) of control (black) and myd88 knockdown embryos (red) iv injected with B. cenocepacia K56-2. (TIF) [file ppat.1006437.s007.tif]

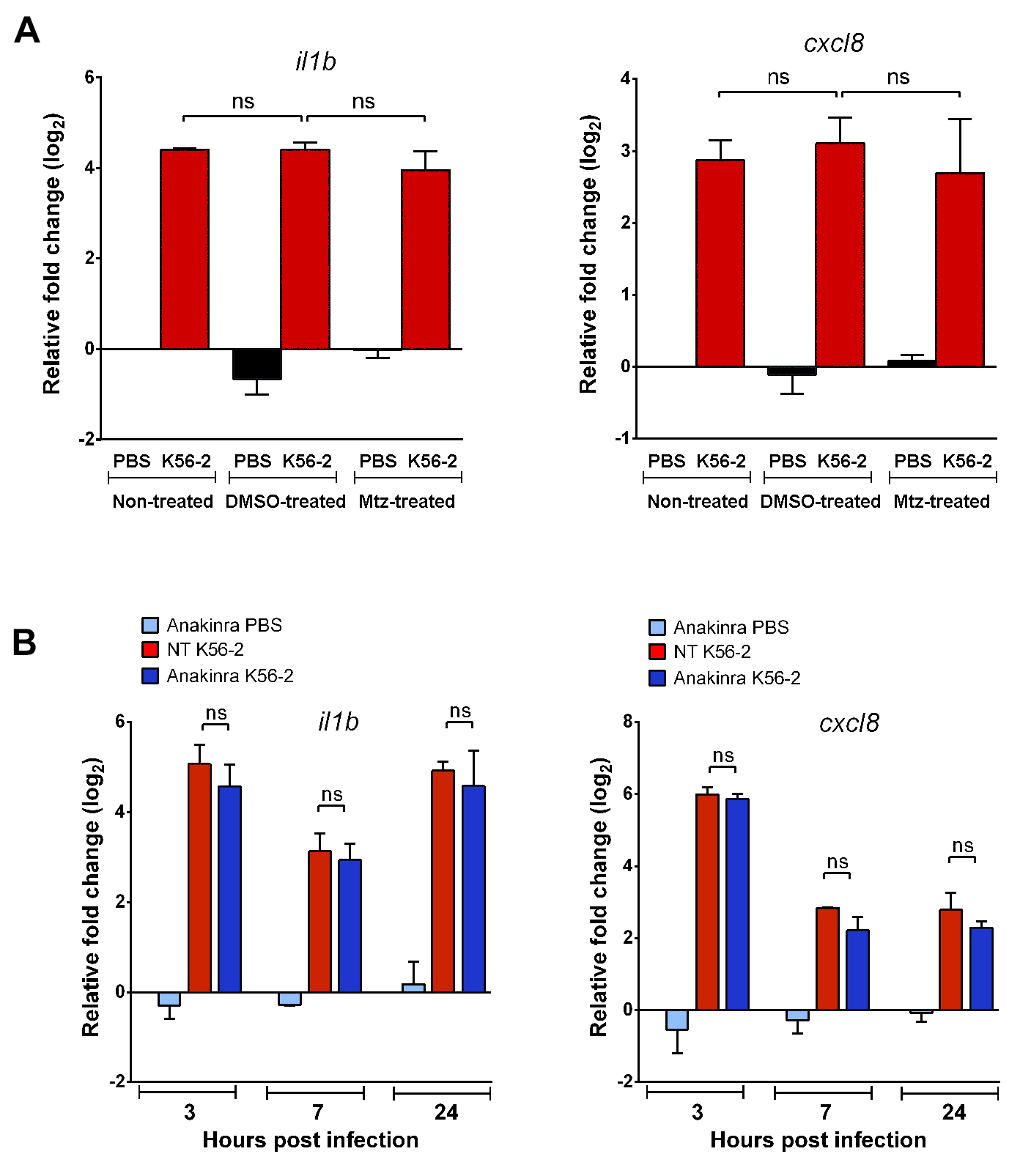

Supplement: S8 Fig — DMSO, Mtz and Anakinra do not affect il1b and cxcl8 expression levels. (A) mpx:GFP embryos were either non-treated or pre-treated at 34 hpf for 15 h with DMSO or 5 mM Mtz. Randomized groups were injected with PBS or B. cenocepacia K56-2 (on average 270 CFU). Graphs show mean relative il1b and cxcl8 gene expression levels, normalized to the PBS-injected non-treated control group at 4 hpi. Error bars represent mean with SEM of two biological replicates. (B) Control and Anakinra-treated mpx:GFP embryos were iv injected with PBS or B. cenocepacia K56-2 (average 120 CFU). Graphs show mean relative il1b and cxcl8 gene expression levels, normalized to the PBS-injected non-treated control group at each time point. Error bars represent mean with SEM of two biological replicates. ns = non-significant. (TIF) [file ppat.1006437.s008.tif]
