# Supplementary material for: Macrophages, but not neutrophils, are critical for proliferation of Burkholderia cenocepacia and ensuing host-damaging inflammation
Source: PLoS Pathog. 2017 Jun 26;13(6):e1006437. doi: 10.1371/journal.ppat.1006437 (PMC5501683; doi:10.1371/journal.ppat.1006437)
Supplement: S1 Table — Morpholinos used in this study. (DOCX) [file ppat.1006437.s009.docx]

**S1 Table.** Related to experimental procedures. Morpholinos used in this study

| Gene | Accession # | Name | Sequence | Target^a^ | [ ]  (mM) ^b^ | Reference |
| --- | --- | --- | --- | --- | --- | --- |
| *pu.1 (spi1)* | ENSDARG00000000767 | tMO_pu.1 | 5'-CCTCCATTCTGTACGGATGCAGCAT-3' | Atg/ 5'UTR | 0,1 | [1] |
|  |  | sMO_E4I5_pu.1 | 5'-GGTCTTTCTCCTTACCATGCTCTCC-3' | E4-I4 | 0,38 |  |
| *cybb (gp91)* | ENSDARG00000056615 | sMO2_cybb | 5'-CATAATCCCGATAGCTTACGATAAC-3' | E1-I1 | 0,8 | [2] |
| *csf3R* | ENSDARG00000045959 | tMO | 5′-GAAGCACAAGCGAGACGGATGCCAT-3 | ATG/5’UTR | 0.5 | [3] |
| *Il1b* | ENSDARG00000098700 | sMO | 5′-CCCACAAACTGCAAAATATCAGCTT-3′ | I2/E3 | 0.5-1 | [4) |
| *myd88* | ENSDARG00000010169 | tMO1-myd88 | 5'- TAGCAAAACCTCTGTTATCCAGCGA-3' | 5’UTR | 0,5 | [5) |
|  |  | sMO2-myd88 | 5'-GTTAAACACTGACCCTGTGGATCAT-3' | E2-I2 | 0,5 | [6] |
|  |  | standard control morpholino | 5'-CCTCTTACCTCAGTTACAATTTATA-3' |  | 0,8 |  |

^a^ For pu.1 and myd88 knockdown, the injection solution was a mix between tMO and sMO. For pu.1, the indicated combination of sMO/tMO allows the specific ablation of macrophages, without affecting neutrophils [7].

^b^ [ ] = concentration; all MOs were injected at 1nL.

References

1. Clay, H., Davis, J.M., Beery, D., Huttenlocher, A., Lyons, S.E., and Ramakrishnan, L. (2007). Dichotomous role of the macrophage in early *Mycobacterium marinum* infection of the zebrafish. Cell Host Microbe *2*, 29–39

2. Yang, C.-T., Cambier, C.J., Davis, J.M., Hall, C.J., Crosier, P.S., and Ramakrishnan, L. (2012). Neutrophils exert protection in the early tuberculous granuloma by oxidative killing of Mycobacteria phagocytosed from infected macrophages. Cell Host Microbe *12*, 301–312.

3. Ellett, F., Pase, L., Hayman, J.W., Andrianopoulos, A., and Lieschke, G.J. (2011). *Mpeg1* Promoter transgenes direct macrophage-lineage expression in zebrafish. Blood *117*, e49–e56.

4. Nguyen-Chi, M., Phan, Q.T., Gonzalez, C., Dubremetz, J.-F., Levraud, J.-P., and Lutfalla, G. (2014). Transient infection of the zebrafish notochord with *E. coli* induces chronic inflammation. Dis. Model. Mech. *7*, 871–882.

5. Sar, A.M. Van Der, Stockhammer, O.W., Laan, C. Van Der, Spaink, H.P., Bitter, W., and Meijer, A.H. (2006). MyD88 innate immune function in a zebrafish embryo infection model. Infect. Immun. *74*, 2436–2441.

6. van der Vaart, M., van Soest, J.J., Spaink, H.P., and Meijer, A.H. (2013). Functional analysis of a zebrafish *myd88* mutant identifies key transcriptional components of the innate immune system. Dis. Model. Mech. *6*, 841–854.

7. Su, F., Juarez, M. a, Cooke, C.L., Lapointe, L., Shavit, J. a, Yamaoka, J.S., and Lyons, S.E. (2007). Differential regulation of primitive myelopoiesis in the zebrafish by Spi-1/Pu.1 and C/ebp1. Zebrafish *4*, 187–199.
