# Supplementary material for: Macrophages, but not neutrophils, are critical for proliferation of Burkholderia cenocepacia and ensuing host-damaging inflammation
Source: PLoS Pathog. 2017 Jun 26;13(6):e1006437. doi: 10.1371/journal.ppat.1006437 (PMC5501683; doi:10.1371/journal.ppat.1006437)
Supplement: S2 Table — Primers used for qRT-PCR experiments. (DOCX) [file ppat.1006437.s010.docx]

**S2 Table.** Related to experimental procedures. Primers used for qRT-PCR experiments.

| Gene | Accession # | Forward qPCR primer | Reverse qPCR primer |
| --- | --- | --- | --- |
| *ppial* | ENSDARG00000042247 | 5’-ACACTGAAACACGGAGGCAAAG-3' | 5’-CATCCACAACCTTCCCGAACAC-3' |
| *mpeg1* | ENSDARG00000055290 | 5’-GTCTTATATCTCCAACAGTCAG-3' | 5’-GATGCCTGGGTAGAATAAAGC-3' |
| *mpx* | ENSDARG00000019521 | 5’-ACCATTGCGAACGTCTTTGC-3' | 5’-ACTGGGAAACTGAGGATGGTTC-3' |
| *cxcl8* | ENSDARG000000104795 | 5’-TGTGTTATTGTTTTCCTGGCATTTC-3' | 5’-GCGACAGCGTGGATCTACAG-3' |
| *il1b* | *ENSDARG00000005419* | 5’-GAACAGAATGAAGCACATCAAACC-3' | 5’-ACGGCACTGAATCCACCAC-3' |
